# Autophagy in the Neuronal Ceroid Lipofuscinoses (Batten Disease)

**DOI:** 10.3389/fcell.2022.812728

**Published:** 2022-02-16

**Authors:** William D. Kim, Morgan L. D. M. Wilson-Smillie, Aruban Thanabalasingam, Stephane Lefrancois, Susan L. Cotman, Robert J. Huber

**Affiliations:** ^1^ Environmental and Life Sciences Graduate Program, Trent University, Peterborough, ON, Canada; ^2^ Centre Armand-Frappier Santé Biotechnologie, Institut National de La Recherche Scientifique, Laval, QC, Canada; ^3^ Department of Anatomy and Cell Biology, McGill University, Montreal, QC, Canada; ^4^ Centre D'Excellence en Recherche sur Les Maladies Orphelines–Fondation Courtois (CERMO-FC), Université Du Québec à Montréal (UQAM), Montréal, QC, Canada; ^5^ Department of Neurology, Center for Genomic Medicine, Massachusetts General Hospital Research Institute, Harvard Medical School, Boston, MA, United States; ^6^ Department of Biology, Trent University, Peterborough, ON, Canada

**Keywords:** autophagosome, autophagy, Batten disease, lysosome, model system, mTOR, neurodegeneration, neuronal ceroid lipofucinosis

## Abstract

The neuronal ceroid lipofuscinoses (NCLs), also referred to as Batten disease, are a family of neurodegenerative diseases that affect all age groups and ethnicities around the globe. At least a dozen NCL subtypes have been identified that are each linked to a mutation in a distinct ceroid lipofuscinosis neuronal (*CLN*) gene. Mutations in *CLN* genes cause the accumulation of autofluorescent lipoprotein aggregates, called ceroid lipofuscin, in neurons and other cell types outside the central nervous system. The mechanisms regulating the accumulation of this material are not entirely known. The *CLN* genes encode cytosolic, lysosomal, and integral membrane proteins that are associated with a variety of cellular processes, and accumulated evidence suggests they participate in shared or convergent biological pathways. Research across a variety of non-mammalian and mammalian model systems clearly supports an effect of *CLN* gene mutations on autophagy, suggesting that autophagy plays an essential role in the development and progression of the NCLs. In this review, we summarize research linking the autophagy pathway to the NCLs to guide future work that further elucidates the contribution of altered autophagy to NCL pathology.

## The Neuronal Ceroid Lipofuscinoses

The neuronal ceroid lipofuscinoses (NCLs) are a family of neurodegenerative diseases that affect all ethnicities and ages but predominantly affect children (Williams, 2011; Mole and Cotman, 2015). Commonly known as Batten disease, the different subtypes are each caused by a mutation in a distinct ceroid lipofuscinosis neuronal (*CLN*) gene (*PPT1/CLN1*, *TPP1/CLN2*, *CLN3*, *DNAJC5/CLN4, CLN5*, *CLN6*, *MFSD8/CLN7*, *CLN8*, *CTSD/CLN10*, *PGRN/CLN11*, *ATP13A2/CLN12*, *CTSF/CLN13*) (Table 1). In addition to these genes, previous research indicates that mutations in potassium channel tetramerization domain containing 7 *(KCTD7)/CLN14* may also cause a subtype of NCL called CLN14 disease (Staropoli et al., 2012) (Table 1). However, recent evidence points towards the majority of CLN14 disease cases being progressive myoclonus epilepsy with an autophagy-lysosome defect but without the classic NCL-type storage material present (Kousi et al., 2012; Metz et al., 2018). In addition, recent work suggests that mutations in TBC1 domain-containing kinase (TBCK)*/CLN15* may cause a new subtype of NCL referred to as CLN15 disease (Table 1) (Liu et al., 2013; Chong et al., 2016; Beck-Wödl et al., 2018). Mutations in *CLN* genes cause the lysosomal accumulation of ceroid lipofuscin, which is an autofluorescent material composed of lipid-protein aggregates (Palmer et al., 1992; Radke et al., 2015). NCL patients experience a variety of clinical symptoms including seizures, progressive loss in vision, movement, and cognitive capability, and premature death (Schulz et al., 2013). The proposed functions of CLN proteins are varied, where some function as lysosomal enzymes, others are predicted to regulate intracellular trafficking or transport across membranes (Cárcel-Trullols et al., 2015). Unfortunately, the cellular roles of most of the CLN proteins are not fully understood, which has motivated the use of a diversity of model systems to study the NCLs, ranging from lower eukaryotic model organisms such as yeast and the social amoeba *Dictyostelium discoideum* to animal models and patient-derived cell lines (Huber et al., 2020; Minnis et al., 2020).

**TABLE 1:** Genes associated with the NCL subtypes, as well as the localizations and molecular functions of proteins encoded by those genes.

Gene	NCL subtype	Disease onset	Localization of protein encoded by gene	Molecular function of protein
*PPT1*	CLN1 disease	Infantile	Lysosomal lumen	Protein thioesterase
		Late-infantile Juvenile	Extracellular	
		Adult		
*TPP1*	CLN2 disease	Late-infantile Juvenile	Lysosomal lumen	Serine protease
			Extracellular	
*CLN3*	CLN3 disease	Juvenile	Lysosomal membrane	Not well defined^a^
			Endosomal membrane	
			Golgi complex	
*DNAJC5*	CLN4 disease	Adult	Perinuclear	Co-chaperone protein
			Lysosome	
			Endo-lysosomal lumen	
			Cytoplasm	
			Plasma membrane	
*CLN5*	CLN5 disease	Late-infantile Juvenile	ER	Glycoside hydrolase^b^
		Adult	Lysosomal lumen	
		Extracellular		
*CLN6*	CLN6 disease	Late-infantile Adult	ER membrane	ER-Golgi protein transport
*MFSD8*	CLN7 disease	Late-infantile	Lysosomal membrane	Not well defined
*CLN8*	CLN8 disease	Late-infantile	ER membrane	ER-Golgi protein transport
			Membranes between ER and Golgi	
*CTSD*	CLN10 disease	Congenital	Lysosomal lumen Extracellular	Aspartyl endopeptidase
		Neonatal		
		Late-infantile Juvenile		
		Adult		
*GRN*	CLN11 disease	Adult	Lysosomal lumen	Not well defined
			Extracellular	
*ATP13A2*	CLN12 disease	Juvenile	Multivesicular bodies Lysosomal membrane	Not well defined
			Membranes of early and late endosomes	
*CTSF*	CLN13 disease	Adult	Lysosomal lumen	Cysteine protease
			Extracellular	
*KCTD7*	CLN14 disease	Infantile	Plasma membrane	Not well defined
*TBCK*	CLN15 disease	Infantile	Perinuclear Centrosome	Rab GTPase-activating protein

a See Cotman and Lefrancois (2021) for a recent review of CLN3-dependent processes.

b See Basak et al. (2021b) for a recent review of CLN5-dependent processes.

Accumulated research across a variety of model systems supports autophagy as a central process disrupted in multiple NCL subtypes. Autophagy is a cellular process that provides cells with energy during periods of stress, degrades unnecessary or dysfunctional intracellular material (e.g., organelles and misfolded proteins), and plays a role in neuroprotection (Tsukada and Ohsumi, 1993; Thumm et al., 1994; Mizushima et al., 2002; Cuervo, 2004; Levine and Klionsky, 2004; Yorimitsu and Klionsky, 2005; Bossy et al., 2008; Glick et al., 2010). In this review, we summarize the impact of loss-of-function mutations in *CLN* genes on the autophagy pathway, and we describe how further studies on this pathway in NCL model systems will establish important mechanistic insights and the potential for autophagy as a therapeutic target. Since research on KCTD7 and TBCK in the context of the NCLs and autophagy is limited, they will not be discussed in this review.

## Autophagy

**FIGURE 1:**
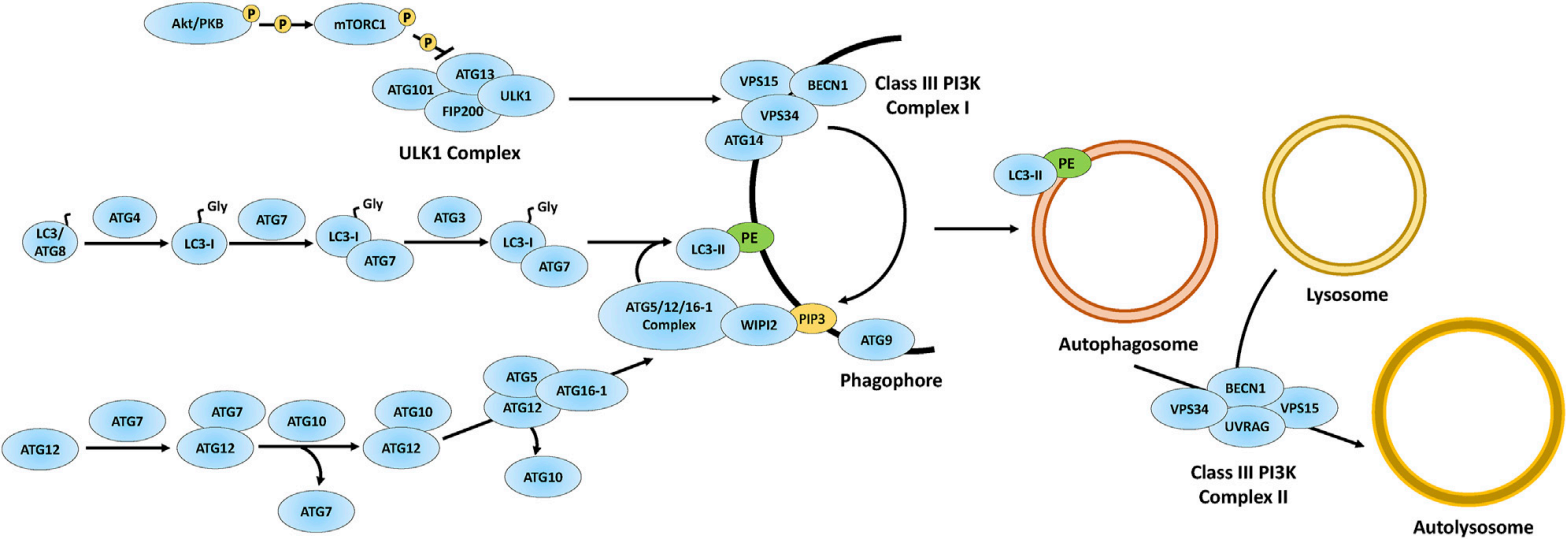
Overview of the autophagy pathway. The autophagy proteins are involved in the formation of autophagosomes *via* phagophore maturation including the ULK1 complex acting on the Class III PI3K complex I to generate PIP3. The autophagy proteins also process LC3 into LC3-I, which matures into LC3-II via attachment to PE on the phagophore. Through phagophore closure, an autophagosome is formed and further matured via autophagosome-lysosomal fusion through the Class III PI3K complex II. Figure concept was adapted from Bento CF. et al. (2016). Akt/PKB, protein kinase B; ATG3, ubiquitin-like-conjugating enzyme ATG3; ATG4, cysteine protease ATG4; ATG5, autophagy protein 5; ATG7, ubiquitin-like modifier-activating enzyme ATG7; ATG9, autophagy protein 9; ATG10, ubiquitin-like-conjugating enzyme ATG10; ATG12, ubiquitin-like protein ATG12; ATG13, autophagy-related protein 13; ATG14, BECN1-associated autophagy-related key regulator; ATG16-1, autophagy-related protein 16-1; ATG101, autophagy-related protein 101; BECN1, beclin-1; FIP200, focal adhesion kinase family interacting protein of 200 kDa; LC3/ATG8, microtubule-associated protein 1A/1B-light chain 3/autophagy-related gene 8; PE, phosphatidylethanolamine; PIP3, phosphatidylinositol-3,4,5-triphosphate; VPS15, vacuolar protein sorting 15; VPS34, vacuolar protein sorting 34; ULK1, UNC51-like autophagy activating kinase 1; UVRAG, UV radiation resistance-associated gene; WIPI2, WD repeat domain phosphoinositide-interacting protein 2.

Autophagic dysregulation has been studied in several neurodegenerative diseases and accumulated evidence indicates it contributes to NCL pathology (Croce and Yamamoto, 2019; Hou et al., 2020; Festa et al., 2021). There are many forms of autophagy, but three main forms in mammals are microautophagy (cytoplasmic material taken up directly by lysosomes/cytoplasmic vacuoles), chaperone-mediated autophagy (selective lysosomal uptake of cytoplasmic macromolecules), and macroautophagy (reviewed in Majeski and Dice, 2004; Li et al., 2012; Feng et al., 2014; Riebisch et al., 2021). Macroautophagy involves the degradation of cytoplasmic macromolecules and organelles during times of cellular stress such as starvation, and this form of autophagy will be the focus of this review. Hereafter, we will use “autophagy” only to refer to macroautophagy. In autophagy, a double-membrane vesicle called an autophagosome forms around intracellular material slated for degradation. The autophagosome is formed through a series of steps that starts with the Unc-51 like autophagy activating kinase 1 (ULK1) complex (Figure 1) (Mizushima, 2010; Quan and Lee, 2013; Bento CF. et al., 2016; Hurley and Young, 2017). The ULK1 complex is composed of four proteins; serine/threonine-protein kinase ULK1, autophagy-related protein 101 (ATG101), autophagy-related protein 13 (ATG13), and focal adhesion kinase family interacting protein of 200 kDa (FIP200), which initiate the formation of the autophagosomal membrane (also known as a phagophore) (Mizushima, 2010; Quan and Lee, 2013; Bento CF. et al., 2016; Hurley and Young, 2017). The ULK1 complex is regulated by mammalian target of rapamycin (mTOR) complex 1 (mTORC1) and protein kinase B (PKB, also known as Akt), whose activation are dependent upon the availability of nutrients, particularly free amino acids (Mizushima, 2010; Mrschtik and Ryan, 2015; Hurley and Young, 2017). Akt is a serine/threonine protein kinase that is activated by phosphatidylinositol-3,4,5-triphosphate (PIP3), which is generated by phosphoinositide-3-kinase (PI3K) signal transduction (Alessi and Cohen, 1998; Dibble and Cantley, 2015). Activated Akt indirectly activates mTORC1 through a series of protein interactions, including dissociation of the tuberous sclerosis complex and GTPase Ras homolog enriched in brain (RHEB) in its GDP form (Dibble and Cantley, 2015). After dissociation occurs, GTP-bound RHEB is formed, which initiates mTORC1 activity (Dibble and Cantley, 2015). mTORC1 is a serine/threonine kinase in the mTOR pathway that is involved in many cellular processes including, but not limited to, autophagy, protein synthesis, and lysosomal biogenesis (Mrschtik and Ryan, 2015; Saxton and Sabatini, 2017). In nutrient-rich conditions, Akt is phosphorylated, which leads to mTORC1 activation (Figure 1) (Mizushima, 2010; Quan and Lee, 2013; Hurley and Young, 2017; Saxton and Sabatini, 2017). Activated mTORC1 then phosphorylates ATG13, which inhibits ULK1 complex kinase activity, consequently preventing autophagosome formation (Mizushima, 2010; Quan and Lee, 2013; Hurley and Young, 2017; Saxton and Sabatini, 2017). Conversely, starvation inhibits mTORC1 activity, which facilitates autophagosomal formation via the ULK1 complex.

The kinase activity of ULK1 in the ULK1 complex causes vacuolar protein sorting (VPS) 34 (VPS34) to associate with the phagophore (Figure 1) (Bento CF. et al., 2016). VPS34 is part of a tetramer complex composed of serine/threonine-protein kinase VPS15, beclin-1 (BECN1), and either BECN1-associated autophagy-related key regulator (ATG14) or UV radiation resistance-associated gene (UVRAG) (denoted as Class III PI3K complex I or Class III PI3K complex II, respectively) (Kang et al., 2011; Quan and Lee, 2013; Bento CF. et al., 2016). The Class III PI3K complex I generates PI3P at the phagophore, which is required for the attachment of microtubule-associated protein 1A/1B-light chain 3 (LC3, also referred to as both LC3-b and autophagy-related protein 8, ATG8, but will be referred to as LC3 from hereon) to the phagophore for autophagosome formation. The Class III PI3K complex II is involved in autophagosome maturation (Figure 1) (Dooley et al., 2014; Bento CF. et al., 2016).

Phagophore closure is regulated by several autophagy proteins (Figure 1) (Kang et al., 2011; Quan and Lee, 2013; Bento CF. et al., 2016). Cysteine protease ATG4 (ATG4) is a cysteine protease that cleaves LC3 to expose the C-terminal glycine which results in the formation of LC3-I (Figure 1) (Bento CF. et al., 2016; Maruyama and Noda, 2018). Ubiquitin (Ub)-like modifier-activating enzyme ATG7 (ATG7) further processes LC3-I through ATP-dependent adenylation, thus forming an ATG7-LC3-I thioester-linked intermediate (Bento CF. et al., 2016; Maruyama and Noda, 2018). Subsequently, LC3 in the ATG7-LC3-I intermediate is transferred and thioester-linked to ubiquitin-like-conjugating enzyme ATG3 (ATG3) (Maruyama and Noda, 2018). Finally, through the actions of the autophagy protein 5 (ATG5)/ubiquitin-like protein ATG12 (ATG12)/autophagy-related protein 16-1 (ATG16-1)/WD repeat domain phosphoinositide-interacting protein 2 (WIPI2) complex, LC3-I in the ATG3-LC3-I intermediate attaches to phosphatidylethanolamine (PE) at the phagophore via an amide bond, forming LC3-II (Dooley et al., 2014; Bento CF. et al., 2016; Maruyama and Noda, 2018).

The ATG5/ATG12/ATG16-1/WIPI2 complex is formed through a series of protein conjugation steps, which includes ATG7 cleaving the C-terminal end of ATG12 to expose a glycine residue (Bento CF. et al., 2016). Like the ATG3-LC3-I intermediate, ATG12 is conjugated to ubiquitin-like-conjugating enzyme ATG10 (ATG10), forming an ATG10-ATG12 intermediate, which is then linked with ATG5. Lastly, the ATG5-ATG12 intermediate binds with ATG16-1 and WIPI2 at the phagophore, which then interacts with ATG16-1 to form a complex at the phagophore (Bento CF. et al., 2016). The ATG5/ATG12/ATG16-1/WIPI2 complex facilitates LC3 PE lipidation at the phagophore, which closes the phagophore leading to the formation of an autophagosome (Figure 1) (Fujita et al., 2008; Romanov et al., 2012; Walczak and Martens, 2013; Bento CF. et al., 2016). Finally, the autophagosome fuses with a lysosome via the aforementioned Class III PI3K complex II, ultimately forming an autolysosome (Figure 1) (see Bento et al., 2016b for detailed mechanism). LC3-II is attached on the cytosolic side of the autophagosome and functions as a receptor for p62 (also referred to as sequestosome 1, SQSTM1), which directs protein cargo for autophagic degradation (Kang et al., 2011; Quan and Lee, 2013; Lippai and Low, 2014). Overall, autophagy proteins are essential for initiating autophagosome formation and generating autolysosomes for autophagic degradation. Previous research indicates that impaired autophagy contributes to the neurodegeneration associated with several neurological diseases, including, but not limited to, Huntington’s disease, Alzheimer’s disease, and Parkinson’s disease (Guo et al., 2018). The examination of these autophagy-related processes, among others, resulted in a considerable amount of research indicating that autophagic dysregulation is a pathological mechanism of the NCLs. In this review, we will discuss the impact of loss-of-function of each *CLN* gene and protein on autophagy using research gathered from multiple model systems, starting from the effects of *CLN* gene mutation on transcription and ending with the roles of CLN proteins on lysosomal reformation.

## The Roles of *CLN* Genes and Proteins in Autophagy

### PPT1/CLN1

**FIGURE 2:**
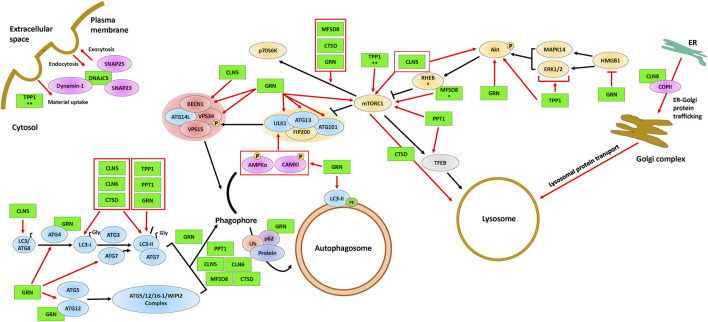
The involvement of CLN proteins in endocytosis, exocytosis, ER-Golgi protein trafficking, autophagosome formation, and lysosome formation. The ULK1 complex (proteins above the yellow background), the Class III PI3K complex I (proteins above the red background), and the autophagy proteins (blue) are required for the formation and maturation of autophagosomes. Some of the CLN proteins (green) affect mTORC1, a protein complex that regulates autophagosome formation and lysosomal biogenesis *via* TFEB. The black arrows indicate the autophagy pathway and red arrows indicate supporting evidence of an effect of a CLN protein on a given autophagic process. Protein overlaps are representative of protein-protein interactions. * indicates the observation was made only for the *D. melanogaster* homolog and ** indicates the observation was made only for the *D. discoideum* homolog. Note that the positions of the CLN proteins depicted in the figure are not necessarily representative of their actual localization in the cell. Please refer to Table 1 for information on the localizations of the different CLN proteins. Akt, protein kinase B; AMPKα, AMP-activated protein kinase α1/2; ATG3, ubiquitin-like-conjugating enzyme ATG3; ATG4, cysteine protease ATG4; ATG5, autophagy protein 5; ATG7, ubiquitin-like modifier-activating enzyme ATG7; ATG9, autophagy protein 9; ATG12, ubiquitin-like protein ATG12; ATG13, autophagy-related protein 13; ATG14L, BECN1-associated autophagy-related key regulator-like; ATG16-1, autophagy-related protein 16-1; ATG101, autophagy-related protein 101; BECN1, beclin-1; CAMKI, calmodulin-dependent protein kinase I; CLN5, ceroid lipofuscinosis neuronal 5; CLN6, ceroid lipofuscinosis neuronal 6; CLN8, ceroid lipofuscinosis neuronal 8; COPII, coat protein complex II; CTSD, cathepsin D; DNAJC5, DnaJ heat shock protein family Hsp40 member C5; Dynamin-1, dynamin-1; ER, endoplasmic reticulum; ERK1/2, extracellular signal-regulated protein kinase 1/2; FIP200, focal adhesion kinase family interacting protein of 200 kDa; GRN, progranulin; HMGB1, high mobility group box 1; LC3/ATG8; microtubule-associated protein 1A/1B-light chain 3/autophagy-related gene 8; LC3-I, processed LC3; LC3-II, lipid-attached membrane-bound LC3; MAPK14, mitogen-activated protein kinase 14; MFSD8, major facilitator superfamily domain containing 8; mTORC1, mammalian target of rapamycin complex 1; P, phosphate; p62, p62/sequestosome 1; p70S6K, p70 S6 kinase; PE, phosphatidylethanolamine; Protein, protein cargo; PPT1, palmitoyl protein thioesterase 1; RHEB, ras homolog enriched in the brain; SNAP23, synaptosomal-associated protein 23; SNAP25, synaptosomal-associated protein 25; TFEB, transcription factor EB; TPP1, tripeptidyl peptidase 1; Ub, ubiquitin; VPS15, vacuolar protein sorting 15; VPS34, vacuolar protein sorting 34. ULK1, UNC51-like autophagy activating kinase 1; WIPI2, WD repeat domain phosphoinositide-interacting protein 2.

**FIGURE 3:**
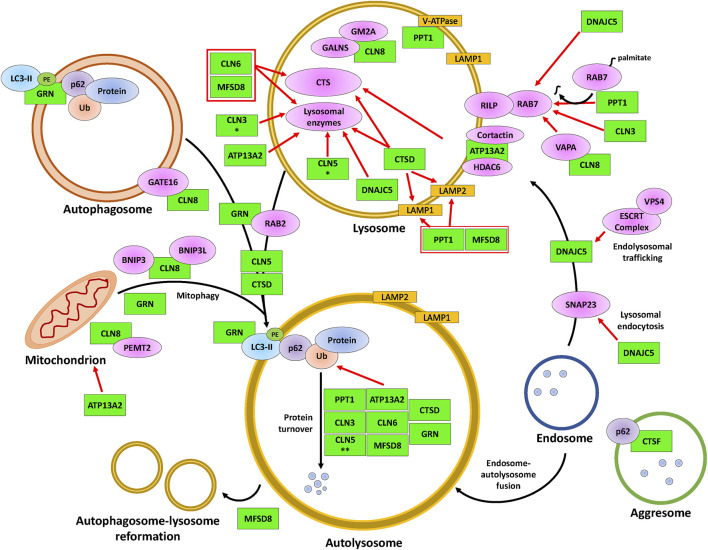
The involvement of CLN proteins in autolysosome formation, cargo degradation, autophagic lysosome reformation, and mitophagy. The CLN proteins (green) modulate various proteins (magenta, yellow, orange) that impact the indicated processes within lysosomes, such as intraluminal acidification and enzymatic degradation, and the fusion of lysosomes with autophagosomes (denoted as autolysosomes) or with mitochondria (known as mitophagosomes, not represented here). While DNAJC5 is involved in regulating endolysosomal trafficking and the fusion between endosomes and autolysosomes, MFSD8 is involved in ALR. The black arrows indicate the autophagic processes starting from autolysosome/mitophagosome formation to ALR. Red arrows indicate supporting evidence of the involvement of a CLN protein within the latter processes of autophagy. Protein overlaps are representative of protein-protein interactions. * indicates the observation was made only for the *D. melanogaster* homolog and ** indicates the observation was made only for the *D. discoideum* homolog. Note that the positions of the CLN proteins depicted in the figure are not necessarily representative of their actual localization in the cell. Please refer to Table 1 for information on the localizations of the different CLN proteins. ATP13A2, polyamine transporting ATPase 13A2; BNIP3, BCL2/adenovirus E1B 19 kDa protein-interacting protein 3; BNIP3L, BCL2/adenovirus E1B 19 kDa protein-interacting protein 3-like; CLN3, ceroid lipofuscinosis neuronal 3; CLN5, ceroid lipofuscinosis neuronal 5; CLN6, ceroid lipofuscinosis neuronal 6; CLN8, ceroid lipofuscinosis neuronal 8; Cortactin, cortactin; CTS, cathepsin B/D/Z; CTSD, cathepsin D; CTSF, cathepsin F; DNAJC5, DnaJ heat shock protein family Hsp40 member C5; ESCRT complex, endosomal sorting complex required for transport complex; GALNS, N-acetylgalactosamine-sulfate; GATE16, GABA type A receptor-associated protein-like 2; GM2A, GM2 ganglioside activator; GRN, progranulin; HDAC6, histone deacetylase 6; LAMP1, lysosomal-associated membrane 1; LAMP2, lysosomal-associated membrane 2; LC3-II, lipid-attached membrane-bound LC3; Lysosomal proteins, PPT1/TPP1/β-glucuronidase/β-hexosaminidase/Saposin C/D; MFSD8, major facilitator superfamily domain containing 8; p62, p62/sequestosome 1; PE, phosphatidylethanolamine; Protein, protein cargo; PEMT2, phosphatidylethanolamine N-methyltransferase 2; PPT1, palmitoyl protein thioesterase 1; RAB2, ras-related protein Rab-2A; RAB7, ras-related protein Rab-7A; RILP, RAB-7A-interacting lysosomal protein; SNAP23, synaptosomal-associated protein 23; Ub, ubiquitin; V-ATPase, vacuolar-type ATPase; VAPA; vesicle-associated membrane protein A; VPS4; vacuolar protein sorting 4.

Palmitoyl protein thioesterase 1 (PPT1) is a protein thioesterase that cleaves S-acetylated palmitate (fatty acid chain linkage via thioester bonds) from substrates and localizes to lysosomes, but also has been detected in synaptosomes, synaptic vesicles, axons, and extracellularly (Table 1) (Hellsten et al., 1996; Lu et al., 1996; Verkruyse and Hofman, 1996; Lehtovirta et al., 2001; Ahtiainen et al., 2003; Kohan et al., 2005; Cárcel-Trullols et al., 2015). Mutations in *PPT1* cause CLN1 disease, an NCL subtype that occurs at the infantile, late-infantile, juvenile, and adult stages of life (Table 1) (Schulz et al., 2013; Mole and Cotman, 2015). The function of PPT1 has been linked to lipid/cholesterol metabolism, exocytosis, endocytosis, and apoptosis, as well as synaptic recycling in neuronal cells (Cho and Dawson, 2000; Ahtiainen et al., 2003; Ahtiainen et al., 2006; Kim et al., 2008; Lyly et al., 2008). In mice, the human PPT1 homolog, PPT1 (Table 2), is involved in the mTOR pathway, which plays an essential role in autophagy (Sardiello et al., 2009; Settembre et al., 2011; Yun et al., 2020). mTORC1 has regulatory roles in lysosomal acidification, lysosomal enzyme activity, and autophagic-lysosome reformation (ALR) (Puertollano, 2014). The mTOR pathway also modulates the activity of transcription factor EB (TFEB), the principal autophagy and lysosomal biogenesis regulator (Di Malta et al., 2019). Specifically, mTORC1 inhibits TFEB through phosphorylation, which prevents nuclear translocation of TFEB thereby inhibiting TFEB and inducing a transcriptional response in nutrient-rich environments (Martina et al., 2012; Roczniak-Ferguson et al., 2012; Napolitano et al., 2018; Di Malta et al., 2019). *Ppt1*-deficiency significantly decreases mTORC1 expression and lysosomal translocation, thus impairing mTORC1 function and altering autophagic signalling (Figure 2) (Rabanal-Ruiz et al., 2017; Yun et al., 2020). Additionally, *Ppt1* expression is increased in mouse C2C12 muscle cells during differentiation, and when knocked down, there is a significant increase in lysosomal pH, as well as an increase in lysosomal-associated membrane glycoprotein (LAMP) 2 (LAMP2) and TFEB expression and activity (Yun et al., 2020). Loss of *Ppt1* in mice also leads to the mislocalization and reduced activity of vacuolar-type ATPase (v-ATPase), which consequently, elevates lysosomal pH and impairs autophagic flux (Figure 3) (Bagh et al., 2017). Furthermore, Overall, these studies suggest that PPT1 regulates lysosomal processes, and ultimately autophagy, by modulating TFEB and mTORC1.

**TABLE 2:** Model organisms used to study the roles of *CLN* genes and proteins in autophagy. Alignments were generated using NCBI, Protein BLAST (default search parameters) and the Uniprot ID, of each homolog as query sequences.

Human protein	Size (aa)	Model organism	Homolog	Uniprot ID	Size (aa)	% identity^b^	% similarity^c^
PPT1	306	*D. melanogaster*	Ppt1	Q9W3C7	314	55	72
		*M. musculus*	PPT1	O88531	306	86	92
TPP1	563	*D. discoideum*	Tpp1A	Q55CT0	600	37	52
		*M. musculus*	TPP1	O89023	562	88	94
CLN3	438	*S. pombe*	Btn1p	Q9US09	396	32	47
		*D. discoideum*	Cln3	Q54F25	421	27	45
		*M. musculus*	CLN3	Q61124	438	86	90
DNAJC5	198	*C. elegans*	DNJ-14	E5QCE7	217	51	68
		*M. musculus*	DNAJC5	P60904	198	99	99
CLN5	358	*D. discoideum*	Cln5	Q553W9	322	30	47
		*M. musculus*	CLN5	Q3UMW8	341	78	86
		*O. aries*	CLN5	A2TJ54	361	86	91
CLN6	311	*M. musculus*	CLN6	Q3U466	308	90	93
		*O. aries*	CLN6	Q1PAG8	310	91	93
MFSD8	518	*D. melanogaster*	Cln7	Q9VS51	546	35	57
		*M. musculus*	MFSD8	Q8BH31	519	82	91
CLN8	286	*M. musculus*	CLN8	Q9QUK3	288	85	90
CTSD	412	*D. discoideum*	CtsD	O76856	383	48	65
		*H. armigera*	CtsD	AYP72766^a^	384	54	68
		*M. musculus*	CTSD	P18242	410	83	91
GRN	593	*M. musculus*	GRN	P28798	589	75	84
ATP13A2	1,180	*C. elegans*	CATP-6	F5GUA7	1,207	40	57
		*D. melanogaster*	DmeI/Anne	L0MLL1	1,290	37	54
		*D. rerio*	Atp13a2	A1A5E5	1,170	52	67
		*O. latipes*	Atp13a2	H2L3J0	1,143	50	65
		*M. musculus*	ATP13A2	Q9CTG6	1,169	85	90

a NCBI/GenBank identifier.

b Exact amino acid match.

c Similar amino acid match (e.g., both polar).

The fruit fly, *Drosophila melanogaster*, encodes a homolog of human PPT1, denoted as Ppt1 (Table 2). In *D. melanogaster*, *Ppt1* and phosphatidylserine decarboxylase (*Psd*) interact at the gene level, thus suppressing the activity of both genes and negatively impacting retinas (Midorikawa et al., 2010). Psd is a protein that generates PE through a decarboxylation reaction with phosphatidylserine (Vance and Guergana, 2013). As PE is a key lipid in autophagosome formation, *Psd* knockdown causes reduced levels of the LC3-PE conjugate (Midorikawa et al., 2010). In a *Ppt1*-deficient mouse model, LC3-II levels and autophagosome formation are increased, resulting in an increase in the numbers of autophagosomes (Sarkar et al., 2020; Yun et al., 2020). In another study, LC3-II lipidation experiments showed lysosomal inhibition and a decrease in autophagosome accumulation in *PPT1*-deficient human cells treated with autophagy inhibitors (e.g., chloroquine (CQ) or a chloroquine-derived molecule DC661), indicating that loss of *PPT1* inhibits autophagy (Rebecca et al., 2019). In patient-derived *PPT1*-deficient fibroblasts, studies revealed that LC3-II levels did not differ with inhibition of lysosomal acidification in *PPT1*-deficient cells but increased in fibroblasts derived from CLN1 disease patients, suggesting that autophagic vesicle turnover independent of lysosomal acidification is reduced (Sarkar et al., 2020). Patient-derived *PPT1*-deficient fibroblasts also display decreased LC3 colocalization with both LAMP1 and LAMP2, suggesting altered autophagosomal-lysosomal fusion (Figure 3) (Sarkar et al., 2020). In CLN1 disease models, including mice and human fibroblasts, co-localization of ras-related protein (RAB) 7 (RAB7) with LAMP2 is decreased, as well as poor interactions between RAB7 and RAB7-interacting lysosomal protein (RILP) (Figure 3) (Sarkar et al., 2020). RAB7 is a GTPase that plays a role in endo-lysosomal trafficking, autophagosomal-lysosomal fusion, and lysosomal biogenesis (Cantalupo et al., 2001; Jordens et al., 2001; Zhang et al., 2009; Kuchitsu and Fukuda, 2018; Stroupe, 2018). The loss of *PPT1* in human fibroblasts also reveals both suppressed GTPase activity and elevated amounts of RAB7 (Sarkar et al., 2020). COS-1 cells display reduced RAB7 palmitoylation at mutated cysteine residues (Sarkar et al., 2020). Furthermore, the loss of *PPT1* in humans causes elevated S-palmitoylation along with reduced endo-lysosomal localization of RAB7. These data suggest that the function of PPT1 is important for RAB7 localization (Sarkar et al., 2020). Therefore, PPT1 may be linked to autophagy and autophagic-related processes through its interactions with RAB7, which influences autolysosomal formation. Furthermore, these studies indicate that the function of PPT1 in removing palmitate from its substrates is essential for autophagic function.

Loss of *Ppt1* in mice, as well as *PPT1* in various human models (melanoma cells, patient-derived fibroblasts, patient brain tissues), elevates both p62-positive aggregates and Ub-positive proteins (Figure 3) (Atiskova et al., 2019; Rebecca et al., 2019; Sarkar et al., 2020; Yun et al., 2020). p62 influences the movement of cargo delivery to the proteasome, thus impacting the rate of protein turnover (Liu et al., 2016). Since protein delivery is affected, Atiskova et al. (2019) showed that loss of *Ppt1* in mice impairs autophagosomal-lysosomal fusion, which consequently blocks autophagic cargo degradation. In *Ppt1*-deficient mice, autophagic degradation is elevated (Figure 3) (Yun et al., 2020). While autophagy is one mechanism of protein degradation, protein degradation also occurs via the proteasome. Interestingly, proteasome activity is elevated in human fibroblasts with the loss of *PPT1* (Sarkar et al., 2020). Thus, cargo degradation via autophagy is impacted by the loss of *PPT1* and may be compensated via the proteasome. Collectively, work in fruit flies, mice, and humans indicates that PPT1 plays a role in lysosomal biogenesis, autophagosomal and autolysosomal formation, as well as autophagic cargo turnover.

### TPP1/CLN2

Tripeptidyl peptidase 1 (TPP1) is a serine protease that cleaves tripeptides from the N-terminus of proteins and localizes to lysosomes and extracellularly (Table 1) (Sleat et al., 1997; Vines and Warburton, 1998; Lin et al., 2001; Kohan et al., 2005). Mutations in *TPP1* cause late infantile and juvenile-onset forms of NCL known as CLN2 disease (Table 1) (Schulz et al., 2013; Mole and Cotman, 2015). TPP1 has been linked to various cellular processes including apoptosis, endocytosis, and autophagy (Vines and Warburton, 1998; Junaid et al., 2000; Smith et al., 2019). The role of TPP1 in autophagy has been explored in non-human model systems including mice and *D. discoideum*. In mice, loss of the *TPP1* ortholog, *Tpp1* (Table 2), leads to the differential expression of multiple genes associated with autophagy. For example, cathepsin (CTS) genes (*Ctsc/d/h/s/w/z*) and autophagy-related protein 9b (*Atg9b*) are differentially expressed in *Tpp1*-deficient mice (Table 3) (Domowicz et al., 2019). Cathepsins play important roles in autophagy by modulating lysosomal biogenesis, autophagosomal-lysosomal fusion, and autophagic degradation (Yang et al., 2014; Qi et al., 2016; Yadati et al., 2020). In mouse hepatocytes, loss of *Atg9b* suppresses autophagy (Wang et al., 2017; Domowicz et al., 2019). Genes associated with autophagosomal formation and maturation are also differentially expressed in *Tpp1*-deficient mice (Domowicz et al., 2019). For example, there is altered expression of phosphoinositide 3-kinase (phosphatidylinositol-4,5-bisphosphate 3-kinase catalytic subunit gamma*, Pik3cg*), as well as multiple phospholipase C (*Plc*) genes (*Plcb2, Plcl2, Plch2, Plcg2*) (Table 3) (Domowicz et al., 2019). *Rab7b*, which is associated with autophagosome maturation, is also differentially expressed in *Tpp1*-deficient mice (Table 3) (Domowicz et al., 2019). RAB7B is a GTPase that functions in late endosome-trans-Golgi network vesicular trafficking (Bucci et al., 2010; Progida et al., 2010). In U2OS cells, RAB7B interacts with ATG4B, which regulates autophagic degradation (Kjos et al., 2017). ATG4B is involved in autophagosome maturation via LC3 cleavage which RAB7B modulates (Xie et al., 2008; Yang et al., 2015; Kjos et al., 2017). Thus, loss of *Tpp1* in mice leads to the differential expression of proteins associated with autophagy, but further analysis at a protein level is required to validate the connection to autophagy.

**TABLE 3:** Effects of mutations in *CLN* genes on the expression of autophagy-related genes.

Gene	Model organism	Autophagy-related gene	Protein	Protein function	Transcriptional effect
*TPP1*	*M. musculus*	*Rab7b*	Ras-related protein Rab7b	GTPase that regulates endosome-trans-Golgi network vesicular trafficking	Upregulated
		*Atg9b*	Autophagy-related gene 9B	Regulates lipid transport and autophagy	
		*Ctsc*	Cathepsin C	Cysteine protease	
		*Ctsd*	Cathepsin D		
		*Ctsh*	Cathepsin H		
		*Ctss*	Cathepsin S		
		*Ctsw*	Cathepsin W		
		*Ctsz*	Cathepsin Z		
		*Pik3cg*	Phosphatidylinositol-4,5-bisphosphate 3-kinase catalytic subunit gamma	Phosphoinositide kinase	Downregulated
		*Plcb2*	1-phosphtidylinositol 4,5-bisphosphate phospodiesterase beta-2	Phospholipase	
		*Plcl2*	Inactive phopholipase C-like protein 2		
		*Plcg2*	1-phosphatidylinositol 4,5-bisphosphate phosphodiesterase gamma-2		
		*Plch2*	1-phosphatidylinositol 4,5-biphosphate phosphodiesterase eta-2		
*CLN3*	*S. pombe*	*atg3*	Autophagy-related gene 3	Ubiquitin-like transferase	Upregulated
		*atg8*	Autophagy-related gene 8	Phophatidylethanolamine binding protein	
		*sst2*	Ubiquitin specific protease	Peptidase	Downregulated
		*vps28*	ESCRTI complex subunit Vps28	Vesicular trafficking regulator	
		*fis1*	Putative mitochondrial fission protein Fis1	Mitochondrial and outer membrane fission	
		*mcs4*	Response regulator Mcs4	Oxidative stress signal- transducing protein	
		*pek1*	MAP kinase Pek1	Kinase	
*DNAJC5*	*C. elegans*	*E3 ubiquitin ligase*	E3 ubiquitin ligase	Ubiquitin-tagging protein	Downregulated
*CLN6*	*M. musculus*	*Ctsa*	Cathepsin A	Cysteine protease	Downregulated
		*Ctsb*	Cathepsin B		
		*Ctsd*	Cathepsin D		
		*Tpp1*	Tripeptidyl peptidase 1	Serine protease	Downregulated
	*O. aries*	*LAMP1*	LAMP1	Lysosome biogenesis	No change
*MFSD8*	*M. musculus*	*Ctsd*	Cathepsin D	Cysteine protease	Downregulated
		*Ctss*	Cathepsin S		Upregulated
*CTSD*	*M. musculus*	*p62*	p62	Autophagic and ubiquitin-proteasome system receptor	Upregulated
*GRN*	*M. musculus*	*Gabarap*	Gamma-aminobutyric acid receptor-associated protein-like 1	Gamma-aminobutyric acid receptor	Upregulated
		*Tom1*	Target of Myb1 membrane trafficking protein	Protein trafficking	
		*Tfeb*	Transcription factor EB	Lysosome biogenesis and autophagy regulating protein	
		*Atg7*	Ubiquitin-like modifier-activating enzyme ATG7	Ubiquitin-like modifier activating protein	Upregulated (with GRN addition)
*ATP13A2*	*H. sapiens*	*SYT11*	Synaptotagmin 11	Calcium membrane trafficking and ubiquitin substrate protein	Downregulated

The social amoeba *D. discoideum* presents a unique model system for studying the role of TPP1 in autophagy since it is the only classical lower eukaryote model that encodes a *TPP1*-like gene (Huber, 2016). In fact, the *D. discoideum* genome encodes six homologs of human TPP1, denoted as Tpp1A, Tpp1B, Tpp1C, Tpp1D, Tpp1E, and Tpp1F (Huber et al., 2020). Of these six proteins, only Tpp1A, Tpp1B, and Tpp1F have been studied. Tpp1A localizes to lysosomes, while Tpp1B and Tpp1F localize to the Golgi complex (Phillips and Gomer, 2015; Stumpf et al., 2017). Tpp1F also localizes to the endoplasmic reticulum (ER) and lysosomes (Stumpf et al., 2017). Of the three TPP1-like proteins studied in *D. discoideum*, only Tpp1A has so far been shown to influence processes related to autophagy (Table 2) (Phillips and Gomer, 2015). Loss of *tpp1A* in *D. discoideum* reduces cell proliferation, decreases the viability of cells in autophagy-stimulating media (i.e., media lacking arginine and lysine), causes cells to develop precociously, and impacts the development of cells in the presence of the autophagy inhibitors ammonium chloride and CQ (Phillips and Gomer, 2015; Smith et al., 2019). Reduced cell proliferation and precocious development are key indicators of aberrant autophagy in *D. discoideum* and can be explained by the inability of cells to either sense nutrients in their environment or process nutrients that have been internalized (Mesquita et al., 2017). During growth, the outcome is reduced proliferation, while during development, this defect prematurely places cells in a starved state and causes them to progress through the developmental program faster. *tpp1A* knockdown also increases phagocytosis suggesting *tpp1A*-deficient cells may increase the uptake of material to compensate for their reduced ability to break down internal reserves (Smith et al., 2019). Overall, the loss of *tpp1A* displays phenotypes of altered autophagy, however, further experimentation is needed to reveal the molecular basis of the relationship between *tpp1A* and autophagy.

Work in humans has provided support for a role for TPP1 in autophagy where *TPP1*-deficient fibroblasts display reduced LC3-II when starved or when exposed to lysosomal inhibitors (Figure 2) (Vidal-Donet et al., 2013). Consistent with this finding, LAMP1 is also decreased during starvation in *TPP1*-deficient fibroblasts (Vidal-Donet et al., 2013). Conversely, Van Beersel and others suggested that *TPP1*-deficiency does not affect autophagosome formation since they observed normal conversion of LC3-I to LC3-II (Van Beersel et al., 2013). As the Akt-mTOR pathway prevents autophagosome biogenesis via negatively regulating autophagy, Vidal-Donet and others showed that loss of *TPP1* enhances the levels of phosphorylated Akt (Figure 2) (Jung et al., 2010; Vidal-Donet et al., 2013). Also shown in *D. discoideum*, loss of *tpp1A* may affect mTOR signalling (Figure 2) (Smith et al., 2019). In addition, *TPP1*-deficient fibroblasts also display increased expression of two mitogen-activated protein kinases (MAPKs), including mitogen-activated protein kinase 14 (MAPK14) and extracellular signal-regulated kinase (ERK1/2), both of which modulate the Akt-mTOR pathway (Figure 2) (Vidal-Donet et al., 2013). Finally, mTOR-mediated autophagy is often triggered by cellular stress such as an increase in reactive oxygen species (e.g., peroxide and superoxide radicals) (Spencer et al., 2009; Jung et al., 2010). *TPP1*-deficient fibroblasts demonstrate poor catalase activity leading to increased superoxide and peroxide levels, which may be another explanation for the impaired autophagy in CLN2 disease (Vidal-Donet et al., 2013). Overall, these findings show that TPP1 impacts processes mediated by the mTOR pathway including lysosomal biogenesis and signalling that regulates autophagy.

In a separate study, loss of *atg16* (the *D. discoideum ATG16* homolog) (increases Tpp1F) or both autophagy protein 9 (*atg9*) and *atg16* (decreases Tpp1B, Tpp1C, and Tpp1F) alters Tpp1 protein levels in *D. discoideum* (Xiong et al., 2021). Atg9 is a transmembrane protein that localizes to small vesicles and is thought to play a role in membrane trafficking to autophagosomes (Calvo-Garrido et al., 2010; Tung et al., 2010). *atg9*-deficiency disrupts growth, phagocytosis, and multicellular development (Tung et al., 2010; Yamada and Schaap, 2019). ATG16 is thought to link autophagy to the Ub-proteasome system and loss of *atg16* impacts growth, pinocytosis, phagocytosis, and multicellular development (Xiong et al., 2015; Xiong et al., 2018). These data, combined with work on Tpp1A, suggest that Tpp1 proteins in *D. discoideum* may play important roles in membrane trafficking to autophagosomes (Atg9) or linking autophagy to the Ub-proteasome system (Atg16). In total, work in *D. discoideum*, mice, and humans suggests multiple roles for TPP1 in the autophagy pathway, including lysosomal biogenesis, autophagic vesicle trafficking, and cargo degradation.

### CLN3

Mutations in *CLN3* cause a juvenile-onset form of NCL that is the most common subtype of the disease (Table 1) (The International Batten disease Consortium, 1995; Schulz et al., 2013; Mole and Cotman, 2015). The CLN3 protein contains multiple transmembrane domains and localizes primarily to compartments of the endo-lysosomal pathway and the Golgi complex in model organisms (e.g., yeast and *D. discoideum*) and a variety of mammalian cell lines (Table 1) (Järvelä et al., 1998; Haskell et al., 1999; Kida et al., 1999; Kremmidiotis et al., 1999; Chattopadhyay et al., 2003; Ezaki et al., 2003; Persaud-Sawin et al., 2004; Metcalf et al., 2008; Codlin and Mole, 2009; Kama et al., 2011; Getty et al., 2013; Tecedor et al., 2013; Oetjen et al., 2016; Mirza et al., 2019). The precise function of CLN3 is unknown but it has been linked to a variety of cellular processes including intracellular trafficking and protein secretion (Table 1) (Fossale et al., 2004; Luiro et al., 2004; Luiro et al., 2006; Metcalf et al., 2008; Uusi-Rauva et al., 2012; Tecedor et al., 2013; Schultz et al., 2014; Kovacs et al., 2015; Huber, 2017; Yasa et al., 2020). For a recent review on the current understanding of CLN3 function, see Cotman and Lefrancois (2021).

Research in various model organisms further bolsters the evidence pointing to the importance of CLN3 function in autophagy. For example, work on the *Schizosaccharomyces pombe* (yeast) homolog of human CLN3, Btn1p (Table 2), used synthetic genetic array analyses to show that a mutant version of *btn1* harbouring a mutation equivalent to the most common pathogenic mutation in CLN3 disease, has positive and negative genetic interactions with genes associated with autophagy and mitophagy, respectively (Table 3) (Gachet et al., 2005; Minnis et al., 2021). Mitophagy is another form of autophagy that selectively degrades mitochondria (Youle and Narendra, 2011). Work in another model organism, *D. discoideum*, also supports a role for CLN3 in autophagy. The *D. discoideum* genome encodes a single CLN3-like protein denoted as Cln3 (Table 2) (Huber et al., 2014). In *D. discoideum*, Cln3 localizes to the Golgi complex, contractile vacuole (CV) system, and compartments of the endocytic pathway (e.g., endosomes, lysosomes) (Huber et al., 2014; Huber, 2017; Huber et al., 2016). During the growth phase of the life cycle, loss of *cln3* increases endo-lysosomal pH (Huber and Mathavarajah, 2019). Consistent with the localization of Cln3 to the CV system, which regulates osmoregulation, loss of *cln3* affects the ability of cells to respond to osmotic stress (Mathavarajah et al., 2018). Intriguingly, this phenotype is exaggerated in the presence of the autophagy inhibitor ammonium chloride suggesting a role for Cln3 in autophagy (Mathavarajah et al., 2018). Loss of *cln3* in *D. discoideum* causes cells to develop precociously, which is a common phenotype of *D. discoideum* mutants with impaired autophagy that can be explained by the inability of cells to either sense nutrients in their environment or process nutrients that have been internalized (Huber et al., 2014; Mesquita et al., 2017). Loss of *cln3* also affects the expression, activity, and secretion of several lysosomal enzymes (Figure 3) (Huber, 2017; Mathavarajah et al., 2018; Huber and Mathavarajah, 2019). While the loss of *cln3* displays altered autophagy like *tpp1A* in *D. discoideum*, further investigation is required to provide additional support to validate its connection to autophagy.

Notably, reduced trafficking and levels of lysosomal enzymes have also been reported in mammalian CLN3 disease models (Fossale et al., 2004; Metcalf et al., 2008; Appu et al., 2019; Schmidtke et al., 2019). In mice and human cellular models, early-stage defects in the autophagy pathway have been described. In mice and a murine cerebellar cell model harbouring the most common mutation observed in CLN3 disease patients (*Cln3* ^∆ex7/8^) (Table 2), an accumulation of LC3-positive autophagosomes/autolysosomes is detectable at an early disease stage, even prior to measurable accumulation of lysosomal storage material (Cao et al., 2006). Evidence in these models and in patient cells suggests the accumulation of autophagosomes/autolysosomes arises from reduced autophagic degradation (Figure 3) (Cao et al., 2006; Chang et al., 2011; Vidal-Donet et al., 2013; Lojewski et al., 2014; Chandrachud et al., 2015; Petcherski et al., 2019). The mechanisms causing reduced autophagic degradation upon *CLN3*-deficiency are incompletely understood but may be linked to altered RAB7-mediated trafficking and/or altered Ca^2+^ signalling (Figure 3) (Huber et al., 2014; Chandrachud et al., 2015; Petcherski et al., 2019; Yasa et al., 2020; Yasa et al., 2021). Thus, restoration of autophagic degradation in *CLN3*-deficiency models has emerged as a promising therapeutic target (Chang et al., 2011; Chandrachud et al., 2015; Hong et al., 2016; Palmieri et al., 2017; Petcherski et al., 2019; Kinarivala et al., 2020). Interestingly, in one study that utilized a human retinal pigment epithelial cell line and siRNA knockdown of *CLN3*, an enhancement in autophagic degradation was reported (Zhong et al., 2020). While further work is needed to better understand these findings from those previously described, a possible explanation is in the different model systems and mechanisms of inducing *CLN3*-deficiency, which led to an acute depletion of CLN3 in the knockdown system, versus chronic *CLN3*-deficiency induced by gene mutation/knockout systems. Thus, accumulated evidence indicates that CLN3 is associated with the autophagy pathway by affecting the expression and activity of lysosomal enzymes, as well as modulating vesicular trafficking and autophagic degradation.

### DNAJC5/CLN4

Mutations in DnaJ heat shock protein family Hsp40 member C5 (*DNAJC5*) cause an adult-onset form of NCL known as CLN4 disease, which is also referred to as Kufs or Parry disease (Table 1) (Schulz et al., 2013; Mole and Cotman, 2015). The *DNAJC5* gene encodes a cytosolic vesicle-associated co-chaperone that localizes to vesicles around the nucleus, lysosomes, endo-lysosomes, synaptic vesicles, the cytoplasm, and the plasma membrane (Table 1) (Tobaben et al., 2001; Benitez and Sands, 2017; Xu et al., 2018; Lee et al., 2021). DNAJC5 regulates vesicular fusion complexes such as synaptosomal-associated protein (SNAP) 25 (SNAP25), a key protein involved in synaptic vesicle/plasma membrane fusion, and the SNAP receptor (SNARE) protein, which controls synaptic activity (Selak et al., 2009; Burre et al., 2010; Lau et al., 2010; Mohrmann et al., 2010; Sharma et al., 2012; Zhang et al., 2013). Mice lacking *Dnajc5*, the mouse homolog of human *DNAJC5* (Table 2), display progressive neurodegeneration, which is thought to be a result of defective SNAP25 function (Chandra et al., 2005; Sharma et al., 2012). Research in *Dnajc5*-deficient mice shows that SNAP25 is misfolded but is still functionally active, thereby impairing the assembly of SNARE (Tobaben et al., 2001; Chandra et al., 2005; Sharma et al., 2012). Furthermore, unfolded SNAP25 degradation requires the DNAJC5/heat shock cognate 71 kDa protein (HSC70)/small glutamine-rich tetratricopeptide complex to avoid automatic degradation by the proteasome. In *Dnajc5*-deficient mice, the lack of DNAJC5 results in the removal of unfolded functionally active SNAP25 before its refolding by the aforementioned complex thus impairing SNARE complex assembly and function (Tobaben et al., 2001; Tobaben et al., 2003: Chandra et al., 2005; Cadieux-Dion et al., 2013). This work indicates that DNAJC5 plays a role in endo-lysosomal trafficking, which indirectly links its role to autophagy. In mice and humans, SNAP25 and dynamin-1 interact with DNAJC5 to regulate exocytosis and endocytosis, which are processes associated with the autophagy pathway (Figure 3) (Rozas et al., 2012; Sharma et al., 2012; Zhang et al., 2012). Dynamin-1 modulates endocytosis through oligomer formation at vesicles where it invaginates, pinches, and then separates the vesicle from the cellular membrane through GTPase activity (Sweitzer and Hinshaw, 1998). Additionally, in mice, loss of *Dnajc5* decreases dynamin-1 levels (Zhang et al., 2012; Zhang and Chandra, 2014). As dynamin plays an indispensable role in endocytosis, DNAJC5 may also function in endocytosis. Thus, DNAJC5 may be involved in autophagy through its regulation of dynamin-1 (Rozas et al., 2012; Zhang et al., 2012; Zhang and Chandra, 2014).

In human cells, DNAJC5 is also implicated in the secretion of α-synuclein (α-Syn) through misfolding-associated protein secretion (Lee et al., 2021). In HEK293T cells, DNAJC5 localizes to lysosomes and endo-lysosomes. Analyses between DNAJC5 and endo-lysosomes reveal both elevated DNAJC5 and enhanced vesicular endo-lysosomal association with misfolded proteins. This suggests that DNAJC5 modulates endo-lysosomal function through misfolded protein clearance, which occurs via the endosomal sorting complex required for transport (ESCRT) complex (Lee et al., 2021). The ESCRT complex traffics endo-lysosomal-related proteins to endo-lysosomes to undergo microautophagy (Sahu et al., 2011; Zhu et al., 2017; Lee et al., 2021). Within a HEK293T ESCRT model lacking VPS, the trafficking of DNAJC5 and endo-lysosomal misfolded proteins like α-Syn is reduced and not present at all, respectively (Figure 3) (Lee et al., 2021). This supports other research that found that HSC70 is essential for microautophagy, which further confirms that DNAJC5 is a chaperone specific to HSC70 (Sahu et al., 2011; Lee et al., 2021) In another study, primary fibroblasts carrying the DNAJC5 p. L115R point mutation display elevated SNAP23 protein levels, which impacts SNAP/SNARE lysosomal endocytosis (Figure 3) (Benitez and Sands, 2017). Additionally, mouse fibroblasts harbouring the DNAJC5 p. L115R mutation show increased activity of lysosomal enzymes (β-glucuronidase, β-hexosaminidase, PPT1) intracellularly and extracellularly (Figure 3) (Benitez and Sands, 2017). DNAJC5 aggregation, as revealed by the p. L115R mutation, is normally removed and degraded through the autophagy-lysosomal pathway via autolysosomes. However, in CLN4 disease, the degradation of DNAJC5 is drastically increased. Similarly, mice fibroblasts carrying the DNAJC5 p. L115R mutation and treated with lysosomal and autophagy inhibitors display reduced breakdown and removal of DNAJC5 (Benitez and Sands, 2017). Moreover, DNAJC5 p. L115R mutant human primary fibroblasts showed reduced RAB7 levels, which is indicative of elevated autophagosome-lysosome fusion (Benitez and Sands, 2017). This suggests that the autophagic and lysosomal dysfunction seen in CLN4 disease is likely influenced by the improper aggregation and localization of mutated DNAJC5. Overall, DNAJC5 is associated with autophagy through endo-lysosomal trafficking and autophagy-related vesicular fusion events.

Finally, *Caenorhabditis elegans* (worm) encodes a homolog of human DNAJC5 denoted as DNJ-14 (Table 2). In worms, loss of *dnj-14* reduces neuronal cell bodies and elevates punctate intracellular content (Kashyap et al., 2014). Transcriptional profiling of *dnj-14* mutants revealed a significant downregulation of Ub genes such as E3 ubiquitin ligases, which is noteworthy since ubiquitination is a key element of autophagy that tags proteins for degradation (Table 3) (McCue et al., 2015). From this work, it would appear that DNAJC5 in worms plays some role in protein tagging for degradation, however further work is required to link these findings to the autophagic pathway. Together, work in worms, mice, and humans supports a role for DNAJC5 in autophagy, including endo-lysosomal trafficking, regulating enzymatic activity, autophagosome-lysosome fusion, and targeting protein cargo for degradation.

### CLN5

Mutations in *CLN5* cause late infantile, juvenile, and adult-onset forms of NCL referred to as CLN5 disease (Table 1) (Cannelli et al., 2007; Xin et al., 2010; Mole and Cotman, 2015). CLN5 encodes a 407 amino acid protein that initially localizes to the ER as a type II transmembrane protein, which is subsequently cleaved by members of the signal peptide peptidase family of proteases to generate a mature 358 amino acid protein (Table 1) (Cárcel-Trullols et al., 2015; Jules et al., 2017). The CLN5 protein is glycosylated and trafficked to the lysosomal lumen but is also present extracellularly (Table 1) (Isosomppi et al., 2002; Moharir et al., 2013; Hughes et al., 2014; Huber and Mathavarajah, 2018a). Its sorting to lysosomes is not fully understood, as it is independent of the mannose-6-phosphate receptor (M6PR) and sortilin, suggesting that it uses another pathway, or that both M6PR and sortilin can be used interchangeably (Schmiedt et al., 2010; Mamo et al., 2012). Research suggests CLN5 has glycoside hydrolase activity and the protein is predicted to participate in a variety of biological processes including, but not limited to, cell proliferation, biometal homeostasis, sphingolipid metabolism, lysosomal pH maintenance, endosome-to-trans Golgi network retrieval of sortilin by modulating RAB7 function and retromer recruitment, and autophagy (Table 1) (El Haddad et al., 2012; Mamo et al., 2012; Grubman et al., 2014; Best et al., 2017; Leinonen et al., 2017; Huber and Mathavarajah, 2018a; Adams et al., 2019; Doccini et al., 2020; Yasa et al., 2021). For a recent review on the current understanding of CLN5 function, see Basak et al. (2021b).

In glioblastoma multiforme, *CLN5* knockdown promotes apoptosis but inhibits cell proliferation, migration, and invasion (Xing et al., 2021). *CLN5* knockdown also inhibits the activation of Akt and the mTOR signalling pathways by decreasing the levels of phosphorylated Akt and mTOR (Figure 2). From this, it may be suggested that CLN5 is involved in modulating an autophagic response via phosphorylation events related to mTOR.

Like TPP1, *D. discoideum* is the only lower eukaryotic model organism to encode a homolog of human CLN5 denoted as Cln5 (Table 2) (Huber, 2016). In *D. discoideum*, Cln5 localizes to the ER, punctate distributions in the cytoplasm, cell periphery, CV system, and extracellularly (Huber and Mathavarajah, 2018a; Huber and Mathavarajah, 2018b). Secretion of Cln5 is reduced when autophagy is inhibited pharmacologically (e.g., ammonium chloride, CQ) or genetically (loss of autophagy protein 1 (*atg1*) or *atg9*) (Huber and Mathavarajah, 2018b; McLaren et al., 2021). In addition to osmoregulation, the CV system in *D. discoideum* also regulates unconventional protein secretion (Sesaki et al., 1997). Thus, these observations indicate that Cln5 is secreted *via* an unconventional pathway linked to autophagy induction.

Loss of *cln5* in *D. discoideum* leads to phenotypes associated with aberrant autophagy. *cln5*-deficiency reduces cell proliferation but has no effect on pinocytosis suggesting that the ability of *cln5*-deficient cells to process internalized nutrients is compromised (McLaren et al., 2021). In addition, the proliferation and viability of *cln5*-deficient cells are severely impacted when cells are grown in autophagy-stimulating media (McLaren et al., 2021). At the molecular level, loss of *cln5* increases autophagic degradation and the basal level of autophagy during growth and increases the numbers of Ub-positive proteins (Figure 3) (McLaren et al., 2021). In addition, loss of *cln5* alters the amounts and activities of several lysosomal enzymes, several of which are Cln5-binding proteins (Figure 3) (Huber and Mathavarajah, 2018a; McLaren et al., 2021). Finally, like *D. discoideum tpp1A* ^*-*^ cells, *cln5*-deficiency causes cells to develop precociously. Loss of *cln5* also compromises the development of cells treated with the autophagy inhibitor ammonium chloride (McLaren et al., 2021). Finally, recent work shows that loss of *atg9* increases the amount of intracellular Cln5 (Xiong et al., 2021). Since Atg9 functions in membrane trafficking to autophagosomes, this finding suggests Cln5 may also play a role in vesicular trafficking within the autophagic pathway (Calvo-Garrido et al., 2010; Tung et al., 2010).

Like many neurodegenerative diseases, defective autophagy is observed in animal models of CLN5 disease (Best et al., 2017; Leinonen et al., 2017; Adams et al., 2019; Doccini et al., 2020). For example, mixed neuronal cell cultures derived from *CLN5*-deficient sheep (Table 2) display decreased autophagy compared to cell cultures from wild-type sheep (Best et al., 2017). The cause of decreased autophagy in these cell lines is not fully understood, however, lysosomes from these cells display decreased acidity. This could result in decreased lysosomal function, which would directly impact the degradation of cargo within autophagosomes by lysosomal enzymes. These observations align with findings from mice, a small animal model that also encodes a homolog of human CLN5 (Table 2). In a rodent model of CLN5 disease, defects in autophagy are observed in the retina (Leinonen et al., 2017). Compared to control mice, *CLN5*-deficiency increases the levels of p62 and BECN1, as well as the ratio of LC3-II to LC3-I (Figure 2 and Figure 3). These data suggest a lack of autophagosome-lysosome fusion in *CLN5*-deficient mice, resulting in the accumulation of these autophagic proteins (Leinonen et al., 2017).

Many cell culture models are currently being used to better understand the mechanisms underlying autophagic defects in CLN5 disease. These include patient-derived fibroblasts, and HeLa and HEK293 cells that have been manipulated genetically using CRISPR/Cas9 to delete *CLN5* or treated with RNAi to reduce the amount of CLN5 in cells (Adams et al., 2019; Yasa et al., 2021). When *CLN5* is knocked down in cells and patient fibroblasts, LC3-II accumulation occurs in non-stressed conditions and amino acid-starved cells can degrade LC3 and p62 (Figure 2) (Adams et al., 2019). This suggests an upregulation of autophagy, with no defects in degradation. Supporting this observation, with the use of mRFP-GFP-LC3 probe in non-starved cells, more red fluorescence was observed in *CLN5*-deficient patient-derived fibroblasts compared to wild-type cells, suggesting efficient fusion between autophagosomes and lysosomes (Adams et al., 2019). Results from this work diverges from work previously done in both the sheep and mouse models of CLN5 disease. In contrast, work done in *CLN5*-deficient HeLa cells generated using CRISPR/Cas9 shows significantly reduced red fluorescence in amino acid starved cells using a mTagRFP-mWasabi-LC3 autophagy probe (Yasa et al., 2021). These cells also displayed reduced co-localization of the autophagy marker LC3 with the lysosomal marker LAMP1, as well as reduced perinuclear movement of lysosomes as measured by the lysosomal marker CD63, a process necessary for efficient autophagy (Nakamura and Yoshimori, 2017). Similar results are also observed in human cortical-like glutamatergic neurons that were genetically modified using CRISPR/Cas9-mediated gene editing to prevent *CLN5* expression. These cells display reduced lysosomal function, as well as disrupted lysosomal movement, both of which can have a negative impact on autophagic degradation (Figure 3) (Basak et al., 2021a). It is difficult to resolve conflicting results between cells with reduced expression of CLN5 and those that are completely deficient. Perhaps the difference between an acute knockdown versus a prolonged knockout explains the discrepancy. However, most models show a defect in autophagy and not an increase. Furthermore, studies with knockdown and the loss of *CLN5* show lysosomal enzyme defects, which one would predict should have a negative impact on autophagy (Mamo et al., 2012; Basak et al., 2021a; Yasa et al., 2021). Nonetheless, these findings suggest a role for CLN5 in autophagy. These findings, combined with observations from *D. discoideum*, mice and sheep, suggest that CLN5 plays an important role in autophagy by regulating mTOR signalling, lysosomal function, autophagy-related vesicular trafficking, and autophagosome-lysosome fusion.

### CLN6

Mutations in *CLN6* cause both late infantile and adult-onset forms of NCL referred to as CLN6 disease (Table 1) (Schulz et al., 2013; Mole and Cotman, 2015). The *CLN6* gene encodes an uncharacterized transmembrane protein that resides on the ER membrane and is predicted to play a role in biometal homeostasis and autophagy (Mole et al., 2004; Thelen et al., 2012; Kanninen et al., 2013; von Eisenhart-Rothe et al., 2018). Ovine neural cultures with naturally occurring *CLN6* mutations (Table 2) have been vital to our understanding of the role of CLN6 in the context of lysosomal storage accumulation and autophagy (Tammen et al., 2006). For example, Best and others revealed reduced lysosomal acidification, but no change in *LAMP1* expression, in *CLN6*-deficient ovine cultures (Best et al., 2017) (Table 3). The numbers of autophagic compartments (pre-autophagosomes. autophagosomes, autolysosomes), as well as the size of autophagosomes and autolysosomes in *CLN6*-deficient sheep neural cultures were increased (Best et al., 2017). Similar results were observed in mice, another model organism used to study CLN6 disease (Table 2) (von Eisenhart-Rothe et al., 2018). Retinas from CLN6 disease mice display reduced numbers of LC3-positive autophagosomes, along with lowered colocalization of LC3 to autophagosomes, suggesting that autophagosomal formation is inhibited (Figure 2) (von Eisenhart-Rothe et al., 2018). Lentiviral *CLN6* overexpression in *CLN6*-deficient sheep enhances acidification within lysosomes and lowers cytoplasmic vacuole clearance (Best et al., 2017). Thus, CLN6 appears to play a role in both lysosomal acidification and regulating autophagic structures. This is supported by findings from patient-derived samples that indicate that mutations in *CLN6* impair the turnover of autophagic vacuoles (Figure 3) (Cannelli et al., 2009). Overall, CLN6 appears to play a role in lysosomal acidification and autophagic degradation.

As *CLN6*-deficiency impacts the early components in the autophagic process, its impact also cascades into the degradative process in autophagy. CLN6 is proposed to function in trafficking lysosomal enzymes from the ER to Golgi (Bajaj et al., 2020). Lysosomal fractions isolated from *Cln6*-deficient mice display reduced amounts and activities of PPT1, TPP1, CTSD, and CTSB (Figure 3) (Bajaj et al., 2020). Along with these findings, loss of *Cln6* also causes differential expression of *Tpp1* and several *Cts* genes (*Ctsa, Ctsb, Ctsd*) (Table 3) (Bajaj et al., 2020). In *Cln6*-deficient mouse embryonic fibroblasts (MEFs), there is decreased localization of PPT1 and CTSD with LAMP1 (Bajaj et al., 2020). As the enzymes that are key in autophagic degradation are affected by the loss of *Cln6*-deficiency, the mechanism of targeting proteins for autophagic degradation is compromised as well. In CLN6 disease mice, the levels of autophagic markers, Ub-positive proteins, and p62-positive proteins are elevated (Figure 3) (Thelen et al., 2012; von Eisenhart-Rothe et al., 2018). LC3-II/LC3-I levels are further enhanced, with no effect on p62 levels, with the treatment of the autophagy inhibitor CQ in retinas from CLN6 disease mice (Figure 2) (von Eisenhart-Rothe et al., 2018). Although total p62 levels within the retina are unaltered, p62 accumulation occurred within the cytosol suggesting impaired autophagic degradation (von Eisenhart-Rothe et al., 2018). Combined, data from mice, sheep, and humans shows that CLN6 may play a role in trafficking lysosomal enzymes and cargo to autophagosomes, which facilitates autophagic degradation.

### MFSD8/CLN7

Mutations in major facilitator superfamily domain containing 8 (*MFSD8*) cause a late infantile-onset form of NCL referred to as CLN7 disease (Table 1) (Schulz et al., 2013; Mole and Cotman, 2015). MFSD8 is a lysosomal transmembrane protein with unknown function but is predicted to function as a transporter (Pao et al., 1998; Siintola et al., 2007). *D. melanogaster* encodes a homolog of human MFSD8 denoted as Cln7 (Table 2) that participates in autophagy through the mTOR pathway (Connolly et al., 2019). In fruit flies, Cln7 co-localizes and interacts with Rheb (Figure 2) (Connolly et al., 2019). RHEB is a GTPase that interacts with and activates mTORC1 (Yang et al., 2017). The interaction of RHEB with mTORC1 is noteworthy since RHEB anchors mTORC1, as well as the Ragulator complex, on the cytosolic face of lysosomal membranes (Rogala et al., 2019). The regulation of autophagic processes is controlled by various mechanisms including TFEB activation and mTORC1 inactivation (Puertollano, 2014). mTORC1 inactivation is known to initiate autophagosome formation furthering the process of autophagy (Puertollano, 2014). In *Cln7*-deficient fruit flies, mTORC1 activity is affected thus altering autophagy (Figure 2) (Connolly et al., 2019). *Mfsd8*-deficiency also impairs the mTOR pathway in mice, another model organism that contains a homolog of human MFSD8 (Table 2), where abolished phosphorylation of p70 S6 kinase (p70S6K) was observed (Figure 2) (Danyukova et al., 2018). p70S6K is a serine-threonine kinase that has various roles within the cell such as growth and is modulated by the mTOR pathway (Bahrami et al., 2014). Furthermore*,* enlarged autolysosomes, as well as impaired ALR, were revealed in *Mfsd8*-deficient mice (Figure 3) (Danyukova et al., 2018). From these findings, it was suggested that the development of lysosomes from autolysosomes were affected due to poor tubular structures, which are protrusions extending from autolysosomes that generate lysosomes when separated from the extension (Chen and Yu, 2013; Munson et al., 2015; Danyukova et al., 2018). Interestingly, the mTOR pathway has roles within autophagy, where elevated activity in the mTOR pathway induces tubular structures in ALR (Yu et al., 2010). Combined, these findings suggest that MFSD8 may associate with RHEB to anchor mTORC1 on the cytosolic side of lysosomal membranes thereby affecting autophagy at various stages including initiating autophagosome formation as well as lysosome formation via ALR.

Features of lysosomal and autophagic dysregulation are also observed in both mice and post-mortem tissues obtained from individuals with CLN7 disease (Brandenstein et al., 2016; Danyukova et al., 2018; Geier et al., 2019; von Kleist et al., 2019). *MFSD8*-deficiency affects lysosomal function as demonstrated by elevated LAMP1 and LAMP2 levels in mice and in tissues from CLN7 disease individuals (Figure 3) (Brandenstein et al., 2016; Geier et al., 2019). In cerebellar granule neuron precursors isolated from *Mfsd8*-deficient mice, lysosomes are increased in size (von Kleist et al., 2019). As lysosomal dysregulation was observed, the loss of *Mfsd8* in mice alters the levels of lysosomal proteases, including *Ctsd* (reduced) and *Ctss* (elevated), which elevates protease activity (Table 3) (Danyukova et al., 2018). Other studies demonstrate altered lysosomal enzyme activity, including enhanced CTSB and CTSZ activity in MEFs, along with both elevated amounts and activity of CTSD in MEFs and tissues derived from CLN7 disease individuals (Figure 3) (Brandenstein et al., 2016; Geier et al., 2019). Interestingly, loss of *Mfsd8* in mice also reduces the levels of other CLN proteins such as PPT1 and CLN5, which are both suggested to play a role in autophagy (Figure 3) (Danyukova et al., 2018; Adams et al., 2019; Atiskova et al., 2019). As most of these lysosomal proteins are involved in autophagy via proteolysis, reduced protein turnover rate is altered in tissues from individuals with CLN7 disease (Geier et al., 2019). This may also be due to the accumulation of Ub-tagged proteins, as well as elevated p62 levels, which is shown in various locations in *Mfsd8*-deficient mouse brains (Figure 3) (Brandenstein et al., 2016). While *Mfsd8*-deficient mice are capable of undergoing autophagy at a cellular level, loss of *Mfsd8* enhances cell death under prolonged nutrient deprivation (Danyukova et al., 2018). Tissues from CLN7 disease individuals also display elevated numbers of autophagosomal and lysosomal compartments, as well as reduced size of lysosomes, suggesting impairments to autophagosomal-lysosomal clearance (Geier et al., 2019). Collectively, work from fruit flies, mice, and humans indicates that MFSD8 participates in the autophagy pathway through the regulation of mTORC1 and protein degradation.

### CLN8

CLN8 disease is a late infantile-onset form of NCL that is caused by mutations in the *CLN8* gene (Table 1) (Passantino et al., 2013; Schulz et al., 2013; Mole and Cotman, 2015; Gao et al., 2018). CLN8 is an ER transmembrane protein that has been linked to lipid synthesis and transport, membrane trafficking, autophagy, and mitophagy (Lonka et al., 2000; Passantino et al., 2013; di Ronza et al., 2018). In mice, which also contains a homolog of human CLN8, CLN8 is an ER to Golgi cargo receptor that is required for lysosomal biogenesis (Table 1 and 2) (Passantino et al., 2013; di Ronza et al., 2018; Bajaj et al., 2020). CLN8 in mice interacts with coat protein complex II (COPII) to help traffic lysosomal enzymes from the ER to the Golgi (Figure 2) (di Ronza et al., 2018). Furthermore, CLN8 also interacts with various CLN proteins (PPT1, TPP1, CTSD) and other lysosomal proteins including GM2 ganglioside activator (GM2A) and N-acetylgalactosamine-sulfate sulfatase (GALNS) (Figure 3) (di Ronza et al., 2018). Whereas GM2A is a protein that is involved in GM2 ganglioside metabolism, GALNS is an enzyme that acts on polyanionic substrates (Wu et al., 1996; Rivera-Colón et al., 2012). Loss of *CLN8* in HeLa cells affects the maturation of lysosomal enzymes, and mice lacking *Cln8* display reduced amounts and activities of lysosomal enzymes (Figure 3) (di Ronza et al., 2018). Overall, it is clear that CLN8 participates in lysosomal biogenesis.

Studies in mice and human cell models link CLN8 function to mitochondria and mitophagy. In COS-1 and HeLa cells, CLN8 interacts with proteins involved in lipid transport, endosomal trafficking, apoptosis, and autophagy/mitophagy (Passantino et al., 2013). Among these proteins, GABA type A receptor-associated protein-like 2 (GATE16), BCL2/adenovirus E1B 19 kDa protein-interacting protein 3 (BNIP3), and BCL2/adenovirus E1B 19 kDa protein-interacting protein 3-like (BNIP3L) are all known and regular interactors of CLN8 that are directly involved in the autophagic process along with Golgi transport, mitophagy, and apoptosis (Figure 3) (Passantino et al., 2013). GATE16 interacts with autophagosomes and plays a critical role in their development, maturation, and transport (Passantino et al., 2013). BNIP3 and BNIP3L function with other proteins to carry out proper autophagic and mitophagic clearance (Passantino et al., 2013). In addition, mitochondria fuse with autophagosomes during mitophagy (Youle and Narendra, 2011). Interestingly, using recombinant CLN8 in COS-1 cells, CLN8 was shown to interact with vesicle-associated membrane protein A (VAPA), a protein involved in lipid metabolism and membrane fusion with potential links to autophagy (Figure 3) (Passantino et al., 2013). VAPA is known to interact with RAB7 as well as the RAB7-RILP complex, both of which are involved in autophagy (Passantino et al., 2013). In summary, there are many CLN8 interactors that modulate autophagy. Collectively, CLN8 participates in autophagy through its role in lysosomal enzyme trafficking as well as its interactions with autophagy-related proteins. However, as with all forms of NCL, more research is necessary to fully identify and understand the scope and involvement of CLN8 in autophagy.

*Cln8*-deficiency in mice also leads to defective phospholipid synthesis, thus altering the lipid composition and transport of mitochondria-associated membranes (MAMs) (Table 2) (Vance et al., 1997). MAMs are microdomains that facilitate the efficient and specific transport of lipids, ions, and signalling molecules from mitochondria to other organelles such as the ER (Wang et al., 2000; Fujimoto et al., 2012; Lynes et al., 2012). MAMs can bind to the ER and co-regulate cellular processes such as metabolism and autophagy (Missiroli et al., 2016; Theurey et al., 2016). In *Cln8*-deficient mice, it is believed that the defective MAMs are a result of alterations in the levels and types of phospholipids in MAMs, as well as the levels and activities of phospholipid synthesizing enzymes such as phosphatidylethanolamine N-methyltransferase 2 (PEMT2) (Figure 3) (Vance et al., 1997). While the exact function of CLN8 in MAMs and autophagy/mitophagy has yet to be determined, it is clear that the dysregulation of MAMs through lipid transport and synthesis plays a role in autophagy-related processes. As such, examining the role of CLN8 in mitochondria may lead to a clearer picture of autophagic dysregulation due to altered lipid synthesis and transport. In summary, these studies support multiple roles for CLN8 in autophagy, including lysosomal biogenesis, lipid metabolism, and the trafficking of autophagic vesicles.

### CTSD/CLN10

Mutations in *CTSD* cause congenital, neonatal, late infantile, juvenile, and adult-onset forms of NCL that are collectively referred to as CLN10 disease (Table 1) (Schulz et al., 2013; Mole and Cotman, 2015; Varvagiannis et al., 2018). CTSD is an aspartyl endopeptidase that is N-glycosylated and undergoes proteolytic processing to form a mature enzyme that localizes to lysosomes and extracellularly (Table 1) (Poole et al., 1973; Decker et al., 1980; Faust et al., 1985; Erickson, 1989; Rijnboutt et al., 1992; Metcalf and Fusek, 1993; Takeshima et al., 1995). CTSD is linked to a variety of cellular processes including apoptosis and autophagy (Johansson et al., 2003; Benes et al., 2008; Hah et al., 2012). The role of CTSD in autophagy has been explored in a variety of model systems including *D. discoideum,* the lepidopteran insect *Helicoverpa armigera,* and mice (Table 2). In *D. discoideum*, loss of *atg9* and *atg16* reduces the intracellular amount of CtsD, suggesting that CtsD may function in membrane trafficking to autophagosomes or linking autophagy to the Ub-proteasome system (Calvo-Garrido et al., 2010; Tung et al., 2010; Xiong et al., 2018; Xiong et al., 2021). In mice myocardial cells, autophagic clearance correlates with increased expression of *Ctsd* (Wu et al., 2017). Like other CLN proteins, mTOR signalling is affected by the loss of *Ctsd* in mice (Ketterer et al., 2020). Although the levels of LAMP2, RAB7, ULK1, and BECN1 are unaltered in *Ctsd*-deficient mice, *Ctsd*-deficiency in a mouse breast cancer model reduces the localization of mTORC1 to lysosomes when cells are placed in amino acid-limiting conditions (Figure 2) (Ketterer et al., 2020; Iwama et al., 2021). The altered localization is thought to be attributed to proteins that function in tethering mTORC1 to lysosomes, including the Ragulator complex (e.g., Raptor is involved in mTORC1 signalling while Raptor and ras-related GTP binding protein C are both involved in mTORC1 lysosomal attachment), however, this is not the case (Sancak et al., 2008; Lawrence and Zoncu, 2019; Ketterer et al., 2020). Rather, markers of impaired autophagy, including increased LC3-II/LC3-I ratios and increased *p62* mRNA levels, are observed under non-starved conditions and all these findings are thought to be due to altered signalling of mTORC1 as phosphorylation of p70S6K is reduced in cells derived from a *Ctsd*-deficient mouse (Figure 2) (Ketterer et al., 2020). Thus, like MFSD8, mTOR signalling is affected by *Ctsd*-deficiency in mice.

In *H. armigera*, macroautophagy triggers CTSD maturation to promote apoptosis (Di et al., 2021). Ctsd has also been linked to autophagy during ovarian follicular atresia in *Nile tilapia* (Sales et al., 2019). In SH-SY5Y cells carrying various mutations linked to CLN10 disease (including catalytically inactive mutations), the formation of pro-CTSD and mature CTSD is affected, resulting in lowered amounts of CTSD or no CTSD protein being produced at all (Crabtree et al., 2014; Bunk et al., 2021). As some of the CTSD mutations cause either enhanced or decreased CTSD activity, the catalytically inactive CTSD SH-SY5Y cell line also displays enhanced CTSB activity (Figure 3) (Crabtree et al., 2014; Bunk et al., 2021). Similar results of reduced levels of mature CTSD and reduced CTSD activity were also observed in SH-SY5Y cells harbouring a heterozygous *CTSD* mutation, as well as in a *Ctsd*-deficient MEF model (Bae et al., 2015; Marques et al., 2020). In addition, while Crabtree et al. (2014) and Bunk et al. (2021) showed that biomarkers of autophagic dysregulation (e.g., changes in LC3 levels, LAMP1, and LAMP2) are not affected by overexpression of wild-type CTSD or mutated CTSD variants, poor colocalization of CTSD with LAMP2 was shown in SH-SY5Y cells harbouring certain CTSD variants (Figure 3). Although Crabtree et al. (2014) and Bunk et al. (2021) determined no changes in key indicators of dysregulated autophagy in SH-SY5Y cells carrying a heterozygous *CTSD* mutation, the levels of p62 and polyubiquitinated proteins were elevated, which resulted in lysosomal substrate accumulation (Figure 3) (Bae et al., 2015). As CTSD activity is lowered in *Ctsd*-deficient MEFs, the addition of recombinant human CTSD corrected the reduced activity (Marques et al., 2020). In another study, high levels of CTSD activate autophagy by increasing acidic autophagic vacuoles, LC3-II formation, and GFP-LC3 puncta in HeLa cells (Figure 3) (Hah et al., 2012). The addition of human CTSD into *Ctsd*-deficient mice recovers various markers of dysregulated autophagy, such as altered levels of p62, LAMP1, LC3-II/LC3-I, and lysosomal enzymes (saposin C/D amounts and β-hexosaminidase activity) (Figure 2 and Figure 3) (Marques et al., 2020). It also reduces lysosomal subunit C of mitochondrial ATP synthase, a component of ceroid lipofuscin (Marques et al., 2020). In a *Ctsd*-deficient mouse model, expression of recombinant human CTSD can correct lysosomal hypertrophy, storage accumulation, and impaired autophagic degradation in the viscera and central nervous system (Figure 3) (Marques et al., 2020). In rat vascular smooth muscle cells, advanced glycation end products (AGEs) promote proliferation and suppress autophagy by reducing the expression of *Ctsd* (Ma et al., 2015). Overexpression of *Ctsd* prevents AGE-mediated suppression of autophagy. Together, these results suggest that mutations in *CTSD* impact autophagy by affecting the localization and function of CTSD and other lysosomal enzymes.

Furthermore, the role of CTSD in autophagy has also been explored in human cancers. For example, in human colon adenocarcinoma cells, *CTSD* expression is increased when cells are treated with the antineoplastic agent, dichloroacetate, which induces autophagy by inhibiting pyruvate dehydrogenase kinase (Gong et al., 2013). *CTSD* expression is also linked to autophagy in nasopharyngeal lymphomas (Hasui et al., 2011). The autophagy inhibitor, lucathone, impairs autophagic degradation and induces apoptotic cell death in breast cancer cells by stimulating the expression of *CTSD* (Carew et al., 2011). In support of this, CTSD is suggested to play a key role in regulating the switch from apoptosis to autophagy (Zheng et al., 2008). In glioblastoma multiforme, *CTSD* is highly expressed in radioresistant clones, which correlates to an increased level of autophagy (Zheng et al., 2020). The CTSD protein level is positively correlated with the autophagy marker LC3-II/I and negatively correlated with p62, In addition, knocking down *CTSD* with small interfering RNA increases radiosensitivity in glioblastoma cells. Inhibiting CTSD increases the formation of autophagosomes but decreases the formation of autolysosomes, indicating that CTSD regulates the radiosensitivity of glioblastoma multiforme by affecting the fusion of autophagosomes and lysosomes (Zheng et al., 2020). Combined, these observations support a role for CTSD in regulating mTOR signalling, lysosomal protein abundance and activity, and the formation of autophagosomes and autolysosomes.

### PGRN/CLN11

Progranulin (PGRN, officially known as GRN) is a soluble glycoprotein that is proteolytically processed into 6 kDa granulins within endo-lysosomes by cathepsins (Table 1) (Toh et al., 2011; Zhou et al., 2017; Mohan et al., 2021). Although its function is not well defined, GRN is known to localize within lysosomes and extracellularly, and has been linked to cell migration, inflammation, and processes related to cancer progression (Table 1) (Zhou et al., 1993; Tian et al., 2016; Daya et al., 2018; Paushter et al., 2018; Voshtani et al., 2019). Mutations in *GRN* cause an adult-onset form of NCL referred to as CLN11 disease as well as frontotemporal lobar dementia in heterozygous individuals (Table 1) (Smith et al., 2012; Schulz et al., 2013; Mole and Cotman, 2015). Loss of *GRN* also impacts autophagy. Currently, the involvement of GRN in autophagy has been explored in mice and humans (Table 2). In murine *Grn*-lacking neuroblastoma neuro 2a cells, genes encoding proteins involved in the trafficking, sorting, and fusion of vesicles related to the autophagy-lysosome pathway (endosomes, lysosomes, autophagosomes) are differentially expressed (Table 3) (Altmann et al., 2016; Elia et al., 2019). For example, gamma-aminobutyric acid receptor-associated protein-like 1 (*Gabarapl1*) and target of Myb1 protein 1 (*Tom1*) are differentially expressed (Elia et al., 2019). GABARAPL1 is involved in autophagosome formation and maturation, cargo sequestration, and autophagic degradation (Okazaki et al., 2000; Chakrama et al., 2010). TOM1 localizes to endosomes and is involved in trafficking endosomal proteins for autolysosome fusion and influencing autophagosome maturation (Okazaki et al., 2000; Chakrama et al., 2010; Tumbarello et al., 2012; Boyer-Guittaut et al., 2014; Manil-Segalen et al., 2014; Szalai et al., 2015). Similarly, the gene encoding for the lysosomal sorting protein receptor sortilin, which functions in directing proteases including CTSD and CTSH towards lysosomes, is also differentially expressed (Canuel et al., 2008; Braulke and Bonifacino, 2009; Elia et al., 2019). In summary, although the loss of *Grn* in mice causes the differential expression of autophagy-related genes, the direct effects of the loss of *Grn* on autophagy needs to be validated at the protein level.

Recent work in mice links GRN to autophagy through the mTOR and AMP-activated protein kinase (AMPK) pathways (Zhou et al., 2019b). Work in yeast showed that AMPK alpha (AMPKα) phosphorylates Ulk1 to activate autophagy (Kim et al., 2011). Additionally, AMPKα regulates autophagosome formation and the fusion of autophagosomes with lysosomes (Jang et al., 2018). In HEK293T cells, the loss of AMPKα1 impairs autophagy during starvation (Jang et al., 2018). In mice, loss of *Grn* increases the expression of genes linked to lysosomal biogenesis, including *Tfeb* (Table 3) (Tanaka et al., 2014). Furthermore, *Grn*-deficient diabetic mice in high-glucose conditions display lowered phosphorylation of AMPKα and calmodulin-dependent protein kinase I (CAMKI), which reduces autophagic initiation (Figure 2) (Zhou et al., 2019b). Increased levels of phosphorylated Akt are observed with GRN supplemented in C57BL/6 J mice diet, as well as a combined treatment of GRN and insulin, which demonstrates that autophagic signalling is affected under certain glucose conditions (Liu et al., 2015). In human podocytes, GRN-supplemented media causes differential phosphorylation of proteins linked to autophagosome formation including ULK1, VPS34, and BECN1 under high-glucose events (Figure 2) (Zhou et al., 2019b). GRN treatment also rescues the altered phosphorylation of AMPKα, mTOR, and p70S6K (Figure 2). Also in human podocytes, when either *AMPKα1* or *AMPKα2* are silenced, the restorative effects of exogenous GRN treatment on autophagy under glucose-rich conditions are lost, indicating that AMPKα1 and possibly AMPKα2 are the proteins that GRN induce its effects within autophagy (Zhou et al., 2019b). When *GRN*-lacking HeLa cells are treated with the mTOR inhibitor rapamycin, restorative effects of p62 accumulation reduction are observed (Altmann et al., 2016) Thus, loss of *GRN* affects autophagy by impacting phosphorylation in the AMPK pathway, which impacts autophagosome formation via VPS and ULK1.

In mice, loss of *Grn* reduces the amount of ATG12 protein and increases the amount of ATG13 protein, which are both involved in autophagosome formation (Figure 2) (Altmann et al., 2016). GRN co-localizes with the ATG5-ATG12 complex, ATG4b, LC3-b, p62, and LAMP1 at structures resembling autophagosomes (Altmann et al., 2016). GRN has also been shown to interact with ATG4b, ATG12, p62, and LC3-II in a variety of human cell lines (HEK293, SH-SY5Y, HeLa) and tissues (bone marrow, brain) (Altmann et al., 2016; Tegeder, 2016). In macrophage cell cultures derived from mouse bone marrow, loss of *Grn* increases the amount of LC3-II and numbers of autophagosomes (Figure 2) (Chang et al., 2017). When *Grn*-deficient bone marrow cultures derived from mice were exposed to *Listeria monocytogenes*, poor inflammatory response and xenophagy were observed (Chang et al., 2017). Loss of *Grn* in mice leads to accumulation of p62 and LC3-II in autophagosomes (Figure 2 and Figure 3) (Zhao et al., 2021). Furthermore, ovalbumin treatment in *Grn*-deficient mice display elevated amounts in p62, LC3-II, and ATG5-ATG12 amounts, which are indicators of accumulation of autophagosomes (Figure 2) (Zhao et al., 2021). p62 accumulation is also observed in HeLa cells lacking *GRN*, which also display reduced basal autophagy (Altmann et al., 2016). GRN also interacts with proteins that are associated with the fusion of lysosomes with autophagosomes. In HEK293T cells, a region of GRN called granulin E, interacts with RAB2A (Figure 3) (Zhao et al., 2021). Intriguingly, like in *Grn*-deficient mice and *GRN*-deficient human fibroblasts*,* knockdown of *RAB2* in human fibroblasts increases the levels of p62 and LC3-II (Zhao et al., 2021). *RAB2*-deficiency in human fibroblasts also reduces the level of GRN (Zhao et al., 2021). Like other *Grn*-lacking mouse models that display autophagic dysregulation, lysosomal dysregulation is apparent as LAMP1 and CTSD protein levels are increased (Tanaka et al., 2014). *Grn*-deficiency in mice also causes differential enrichment of proteolytic enzymes involved in autophagy including TPP1, CTSD, and CTSZ (Figure 3) (Huang et al., 2020). When recombinant human GRN is added into the medium of *Grn*-deficient mouse podocytes under high glucose conditions (to simulate diabetic conditions) or GRN is supplemented into the diets of C57BL/6 J mice, recovery of reduced mitophagy and mitochondrial biogenesis, as well as increased expression of *Atg7* and LC3-II expression, are observed (Figure 2 and Table 3) (Zhou et al., 2015; Guo et al., 2017; Zhou et al., 2019a). Furthermore, GRN supplemented into the diet of mice reduces the amount of p62 (Figure 3) (Zhou et al., 2015; Guo et al., 2017). The autophagy activator trehalose corrects the reduced *GRN* expression and restored the amount of GRN localizing to the cytoplasm in haploinsufficient *GRN* patient-derived primary fibroblasts (Holler et al., 2016). Together, these findings show that *GRN*-deficiency impacts lysosomal enzyme abundance, autophagosome formation, and cargo tagging.

GRN, ATG7, and LC3-II are all elevated in patients with metabolic syndrome (MS), which are a family of diseases linked to dysfunctional metabolic processes leading to both fat accumulation and insulin insensitivity (Li et al., 2014; Rochlani et al., 2017; Regufe et al., 2020). As with *Grn*-lacking mice models, MS patients display similar mRNA and protein profiles of ATG7 and LC3-II (Figure 2 and Table 3) (Liu et al., 2014). These findings of autophagy impairment are associated with an inflammatory response, which modulate autophagy by generating immune cells (e.g., neutrophils, macrophages, and lymphocytes), as well as inducing transcriptional changes in cytokine production (Li et al., 2014; Qian et al., 2017). HaCaT cells are human keratinocytes under inflammatory conditions that demonstrate high endogenous levels of GRN (Tian et al., 2016). Knockdown of *GRN* elevates the levels of β-catenin, which impacts the Wnt/β-catenin signalling pathway (Tian et al., 2016). *GRN*-deficient HaCaT cells display reduced LC3-II levels, and inhibition of the Wnt/β-catenin signalling pathway through IWP-2 treatment elevates the levels of LC3-II affected by *GRN*-deficiency (Tian et al., 2016). Under other inflammatory-based conditions like asthma, recombinant GRN delivered nasally ameliorates indicators of impaired autophagy such as lowering protein levels of LC3, BECN1, and the number of autophagosomes in mice (Liu et al., 2021). Liu et al. (2021) show and postulate that these autophagic indicators may be regulated by GRN via high mobility group box 1 (HMGB1), a protein that is upstream of the MAPK/ERK pathway and is suppressed by GRN, which negatively modulates autophagy (Figure 2). As GRN is known to be a protective biomolecule in the context of several neurodegenerative diseases, GRN may be asserting its protective effect against excitotoxicity within the autolysosomal pathway despite being an extracellular protein (Davis et al., 2021; Terryn et al., 2021). Thus, these findings suggest that GRN regulates autophagy by initiating autophagosome formation, influencing lysosomal protein production, and participating in cargo degradation.

### ATP13A2/CLN12

Mutations in polyamine-transporting ATPase 13A2 (*ATP13A2*) cause a juvenile-onset form of NCL referred to as CLN12 disease, as well as Kufor Rakeb Syndrome, a juvenile-onset form of Parkinson’s disease (Table 1) (Schulz et al., 2013; Mole and Cotman, 2015). In canines, loss of *ATP13A2* causes an adult-onset form of the disease (Bras et al., 2012). *ATP13A2* encodes a P-type ATPase with an unknown function but is predicted to function as an active transporter of inorganic cations (Table 1) (Ramirez et al., 2006; Ramonet et al., 2012; Cárcel-Trullols et al., 2015). ATP13A2 is known to localize to the membranes of endosomes and lysosomes as well as multivesicular bodies (Table 1) (Ramirez et al., 2006; Ramonet et al., 2012; Tsunemi et al., 2014; Cárcel-Trullols et al., 2015). Mutations in *ATP13A2* are known to impair many biological processes, one of which is autophagy. A role for ATP13A2 in autophagy has been documented in multiple model systems. For example, *C. elegans* encodes a homolog of human ATP13A2 referred to as CATP-6 (Table 2). Loss of *catp-6* in *C. elegans* and loss of *ATP13A2* in cells derived from patients with Parkinson’s disease, impact lysosomal function by preventing lysosomal acidification and lysosomal enzyme maturation, which ultimately affects the degradative capacity of lysosomes (Dehay et al., 2012b; Anand et al., 2020). It also reduces the expression of genes required for autophagy and lysosomal function and affects mitochondrial health. In addition, loss of *catp-6* dysregulates iron metabolism, which is consistent with previous work suggesting iron toxicity-induced neurodegeneration in patients carrying mutations in *ATP13A2* (Rajagopalan et al., 2016). In *Danio rerio* (zebrafish), the homolog of human ATP13A2 is denoted as Atp13a2 (Table 2). Loss of *atp13a2* in zebrafish, as well as in *Oryzias latipes* (medaka fish) (Table 2), causes dopaminergic neurodegeneration and lysosomal dysfunction (Matsui et al., 2013; Nyuzuki et al., 2020). *atp13a2*-deficiency also reduces the level of Ctsd in brain tissue and impairs intracellular trafficking suggesting alterations to the autophagy pathway (Figure 3) (Matsui et al., 2013; Nyuzuki et al., 2020). Collectively, these results suggest ATP13A2 is involved in autophagy by modulating lysosomal acidification and biogenesis.

The role of ATP13A2 in autophagy also extends into small animal models, including mice, and humans. Mice deficient of *Atp13a2* (Table 2) display protein trafficking defects and age-related motor dysfunction that is preceded by gliosis, as well as the accumulation of both ceroid lipofuscin and ubiquitinated protein aggregates (Kett et al., 2015). Loss of *Atp13a2* also impairs the degradation capacity of lysosomes in mouse primary neurons leading to the accumulation of α-Syn (Usenovic et al., 2012). Decreased expression of *Atp13a2* is also associated with defective autophagy in midbrain dopaminergic neurons from a 1-methyl-4-phenyl-1,2,3,6-tetrahydropyridine-induced Parkinson’s disease mouse model (Wan et al., 2020). Human fibroblasts carrying *ATP13A2* mutations display reduced degradative capacity due to impaired lysosomal acidification and decreased proteolytic processing of lysosomal enzymes, ultimately reducing the clearance of autolysosomes (Figure 3) (Usenovic et al., 2012; Dehay et al., 2012a). Loss of *ATP13A2* in humans also negatively regulates the expression and post-translational levels of synaptotagmin-11 (SYT11), which leads to lysosomal dysfunction and impaired degradation of autolysosomes (Table 3) (Bento C. F. et al., 2016). Thus, these findings provide additional evidence linking ATP13A2 to autophagy, specifically cargo degradation within autolysosomes.

Other research suggests that ATP13A2 may serve as a novel prognostic biomarker for colon cancer and a potential target for colon cancer therapy due to increased expression of *ATP13A2* linking to shorter overall survival of colon cancer patients (Chen et al., 2020). Conversely, decreased expression of *ATP13A2* decreases tumorigenesis by blocking autophagic degradation (Figure 3). In melanoma and neuroblastoma cell lines subjected to proteotoxic stress, overexpression of full-length ATP13A2, catalytically-inactive ATP13A2, or the N-terminal domain of ATP13A2 reduces the accumulation of Ub-conjugated proteins by serving as a scaffold for trafficking intracellular cargo (Figure 3) (Demirsoy et al., 2017). Conversely, depletion of *ATP13A2* increases the accumulation of Ub-conjugated proteins (Figure 3). Knockdown of *ATP13A2* fragments mitochondria and increases the production of reactive oxygen species in SH-SY5Y neuroblastoma cells and primary cortical neurons from mice (Gusdon et al., 2012). Loss of *ATP13A2* also decreases autophagic degradation but has no effect on the basal levels of the autophagosome marker LC3-II (Figure 2 and Figure 3). Together, these findings suggest that *ATP13A2*-deficiency impacts autophagy by affecting the trafficking and degradation of ubiquitinated proteins.

Thioredoxin-interacting protein activates oxidative stress and inhibits autophagic degradation by inhibiting thioredoxin (Deng et al., 2020). In HEK293 cells, overexpression of thioredoxin-interacting protein induces LC3-II expression but fails to degrade the Ub-binding protein p62, which is an autophagosome cargo protein that targets proteins for degradation by autophagy (Su et al., 2017). Increased expression of thioredoxin-interacting protein also reduces the expression of *ATP13A2*. Intriguingly, overexpression of *ATP13A2* attenuates the impaired autophagic degradation in cells overexpressing thioredoxin-interacting protein. Finally, Wang et al. (2019) used *Atp13a2*-deficient mice, brains from *D. melanogaster* subjected to RNAi against the *D. melanogaster* homolog of *ATP13A2*, *ATP13A2*-null HEK293, and HeLa cells to show that loss of *ATP13A2* impairs autophagosome-lysosome fusion and damages mitochondria by affecting the recruitment of histone deacetylase 6 (HDAC6) and cortactin to lysosomes (Figure 3). Combined, these findings further suggest that ATP13A2 functions in multiple steps of the autophagy pathway including lysosomal biogenesis, autophagosome-lysosome fusion, protein trafficking, autophagic degradation, and mitochondrial homeostasis.

### CTSF/CLN13

CTSF is a cysteine protease that localizes both to lysosomes and extracellularly (Table 1) (Wang et al., 1998; Nägler et al., 1999; Öörni et al., 2004; Kaakinen et al., 2007; Cárcel-Trullols et al., 2015). Mutations in *CTSF* cause an adult-onset form of NCL referred to as CLN13 disease (Table 1) (Schulz et al., 2013; Mole and Cotman, 2015). In *D. discoideum*, loss of *atg16* (increases cysteine protease (Cpr) E (CprE) and CprF or *atg9* and *atg16* (decreases CprD and CprF) affects the intracellular levels of several homologs of human CTSF suggesting that CTSF homologs in *D. discoideum* may function in membrane trafficking to autophagosomes or linking autophagy to the Ub-proteasome system (Calvo-Garrido et al., 2010; Tung et al., 2010; Xiong et al., 2018; Xiong et al., 2021). In HEK293T cells, CTSF localizes to vesicles and co-localizes with LAMP2 (Jerič et al., 2013). Mutant forms of CTSF that are truncated on the N-terminal end form insoluble perinuclear–juxtanuclear aggregates that accumulate within aggresome-like p62-positive inclusions and co-localize with the autophagy marker LC3B, but not with LAMP2 (Figure 3). Aggresomes are cytoplasmic vesicles that contain aggregates of misfolded proteins and p62 modulates autophagic degradation of polyubiquitinated cargo via interactions with LC3 (Johnston et al., 1998; Pankiv et al., 2007). These findings suggest that N-terminally truncated forms of CTSF are cleared via autophagy. While these data do not suggest a direct involvement of CTSF in autophagy, they do show that levels of the protein are regulated by autophagy genes and that the protein interacts with autophagy-related proteins (e.g., LAMP2).

## Conclusion and Future Perspectives

This review highlights the roles of *CLN* genes and proteins in autophagy. Since mutations in *CLN* genes cause neurodegeneration that is associated with compromised autophagy, targeting this essential cellular process may provide new therapeutic opportunities. For example, in CLN3 disease, multiple preclinical studies have shown promise for pharmacologic approaches to stimulate autophagy/autophagic degradation (Chang et al., 2011; Palmieri et al., 2017; Petcherski et al., 2019; Kinarivala et al., 2020; Soldati et al., 2021). Notably, two upcoming clinical trials for CLN3 disease to test compounds that promote TFEB activation, which enhances autophagy, have been announced by Theranexus and the Beyond Batten disease Foundation (BBDF-1010, Investigational New Drug approval announced, beyondbatten.org) and Polaryx (PLX-200, ClinicalTrials.gov identifier: NCT04637282). Compounds/targets that enhance autophagic degradation are also candidates for the treatment of other forms of NCL; in particular, forms that show reduced degradation upon loss of function of the CLN protein (e.g., MFSD8/CLN7, ATP13A2/CLN12). For many of the NCLs caused by lysosomal enzyme deficiencies, pharmacologic approaches leading to enhanced lysosomal biogenesis and stimulation of autophagy also merit further study, since this may partially compensate for the impact of enzyme deficiency on lysosomal function. Further studies are needed that will more thoroughly dissect the mechanisms of autophagic dysregulation for each of the subtypes of NCL, but it is likely that multiple forms of NCL could benefit from common approaches targeting the autophagy pathway. Aberrant autophagy is also linked to other forms of neurodegeneration including Alzheimer, Huntington, and Parkinson disease (Croce and Yamamoto, 2019; Hou et al., 2020; Festa et al., 2021). Therefore, advances towards therapies targeting autophagy are very likely to impact drug development efforts across the NCLs and other neurological diseases. Finally, the NCL research community has generated a variety of experimental models to study the NCLs, ranging from model organisms such as yeast and *D. discoideum*, to small (e.g., mice) and large animal (e.g., dogs, sheep) models, and patient-derived human models (Huber et al., 2020; Minnis et al., 2020). Collectively, these systems can be used to further explore the precise roles of *CLN* genes and proteins in regulating autophagy.

## Glossary

α-Syn: α-synuclein
AGE: advanced glycation end product
Akt/PKB: protein kinase B
ALR: autophagic lysosome reformation
AMPK: AMP-activated protein kinase
atg1: autophagy protein 1
ATG3: ubiquitin-like-conjugating enzyme ATG3
ATG4: cysteine protease ATG4
ATG5: autophagy protein 5
ATG7: ubiquitin-like modifier-activating enzyme ATG7
ATG8: autophagy-related protein 8
atg9: autophagy protein 9
Atg9b: autophagy-related protein 9b
ATG10: ubiquitin-like-conjugating enzyme ATG10
ATG12: ubiquitin-like protein ATG12
ATG13: autophagy-related protein 13
ATG14: BECN1-associated autophagy-related key regulator
Atg16: autophagy-related protein 16
ATG16-1: autophagy-related protein 16-1
ATG101: autophagy-related protein 101
ATP13A2: polyamine-transporting ATPase 13A2
BECN1: beclin-1
BNIP3: BCL2/adenovirus E1B 19 kDa protein-interacting protein 3
BNIP3L: BCL2/adenovirus E1B 19 kDa protein-interacting protein 3-like
CAMKI: calmodulin-dependent protein kinase I
CLN: ceroid lipofuscinosis neuronal
COPII: coat protein complex II
Cpr: cysteine protease
CQ: chloroquine
CTS: cathepsin
CV: contractile vacuole
DNAJC5: DnaJ heat shock protein family Hsp40 member C5
ERK1/2: extracellular signal-regulated protein kinase 1/2
ESCRT: endosomal sorting complex required for transport
FIP200: focal adhesion kinase family interacting protein of 200 kDa
GABARAPL1: gamma-aminobutyric acid receptor-associated protein-like 1
GALNS: N-acetylgalactosamine sulphate
GATE16: GABA type A receptor-associated protein-like 2
GM2A: GM2 ganglioside activator
HDAC6: histone deacetylase 6
HMGB1: high mobility group box 1
HSC70: heat shock cognate 71-kDa protein
KCTD7: potassium channel tetramerization domain containing 7
LAMP: lysosomal-associated membrane glycoprotein
LC3/ATG8: microtubule-associated protein 1A/1B-light chain 3/autophagy-related gene 8
MAM: mitochondria-associated membrane
MAPK: mitogen-activated protein kinase
MEF: mouse embryonic fibroblast
MFSD8: major facilitator superfamily domain containing 8
MS: metabolic syndrome
mTOR: mammalian target of rapamycin
mTORC1: mammalian target of rapamycin complex 1
NCL: neuronal ceroid lipofuscinosis
p62/SQSTM1: p62/sequestosome 1
p70S6K: p70 S6 kinase
PE: phosphatidylethanolamine
PEMT2: phosphatidylethanolamine N-methyltransferase 2
PGRN/GRN: progranulin
PI3K: phosphoinositide-3-kinase
PIP3: phosphatidylinositol-3,4,5-triphosphate
Plc: Phospholipase C
PPT1: palmitoyl protein thioesterase 1
Psd: phosphatidylserine decarboxylase
RAB: ras-related protein
RHEB: ras homolog enriched in the brain
RILP: RAB7-interacting lysosomal protein
SNAP: synaptosomal-associated protein
SNARE: synaptosomal-associated receptor protein
SYT11: synaptotagmin 11
TBCK: TBC1 domain-containing kinase
TFEB: transcription factor EB
TOM1: target of Myb1 protein 1
TPP1: tripeptidyl peptidase 1
Ub: ubiquitin
ULK1: UNC51-like autophagy activating kinase 1
UVRAG: UV radiation resistance-associated gene
V-ATPase: vacuolar-type ATPase
VAPA: vesicle-associated membrane protein A
VPS: vacuolar protein sorting
WIPI2: WD repeat domain phosphoinositide-interacting protein 2.
